# A Review of the Antimalarial, Antitrypanosomal, and Antileishmanial Activities of Natural Compounds Isolated From Nigerian Flora

**DOI:** 10.3389/fchem.2020.617448

**Published:** 2020-12-23

**Authors:** Marzuq A. Ungogo, Godwin U. Ebiloma, Nahandoo Ichoron, John O. Igoli, Harry P. de Koning, Emmanuel O. Balogun

**Affiliations:** ^1^Department of Veterinary Pharmacology and Toxicology, Ahmadu Bello University, Zaria, Nigeria; ^2^College of Medical, Veterinary and Life Sciences, Institute of Infection, Immunity and Inflammation, University of Glasgow, Glasgow, United Kingdom; ^3^School of Health and Life Sciences, Teesside University, Middlesbrough, United Kingdom; ^4^Phytochemistry Research Group, Department of Chemistry, University of Agriculture, Makurdi, Nigeria; ^5^Department of Biochemistry, Ahmadu Bello University, Zaria, Nigeria; ^6^Africa Centre of Excellence for Neglected Tropical Diseases and Forensic Biotechnology (ACENTDFB), Ahmadu Bello University, Zaria, Nigeria

**Keywords:** anti-parasitics, neglected tropical disease (NTD), phytomedicine, Nigeria, drug development

## Abstract

The West African country Nigeria features highly diverse vegetation and climatic conditions that range from rain forest bordering the Atlantic Ocean in the South to the Desert (Sahara) at the Northern extreme. Based on data from the World Conservation Monitoring Center of the United Nations Environmental Protection, Nigeria, with ~5,000 documented vascular plants, ranks amongst the top 50 countries in terms of biodiversity. Such a rich biodiversity implies that the country is rich in diverse secondary metabolites—natural products/unique chemicals produced by the plant kingdom to confer selective advantages to them. Like many tropical countries, Nigeria is also endemic to numerous infectious diseases particularly those caused by parasitic pathogens. These phytochemicals have been exploited for the treatment of diseases and as a result, a new branch of chemistry, natural product chemistry, has evolved, to try to reproduce and improve the therapeutic qualities of particular phytochemicals. In this review, we have compiled a compendium of natural products, isolated from Nigerian flora, that have been reported to be effective against certain protozoan parasites with the aim that it will stimulate interests for further investigations, and give impetus to the development of the natural products into registered drugs. In total 93 structurally characterized natural compounds have been identified with various levels of anti-parasite activity mainly from Nigerian plants. The synthesis protocol and molecular target for some of these natural anti-parasite agents have been established. For instance, the anti-plasmodial compound fagaronine (**7**), a benzophenanthridine alkaloid from Fagara zanthoxyloides has been successfully synthesized in the laboratory, and the anti-trypanosomal compound azaanthraquinone (**55**) elicits its effect by inhibiting mitochondrial electron transfer in trypanosomes. This review also discusses the barriers to developing approved drugs from phytochemicals, and the steps that should be taken in order to accelerate the development of new antiparasitics from the highlighted compounds.

## Introduction

Natural products are broadly defined as chemical entities with pharmacological properties, produced by naturally occurring living organisms such as plants, fungi, bacteria, protists, sponges, including other invertebrates that are present in diverse environments. The prospects of using natural product-based remedies for treating and managing infections can be understood from the perspective of traditional medicines. Historically, natural products have played a key role in fighting infectious diseases across the globe. Archives for such evidence were found in cuneiform engraved on clay tablets from Mesopotamia, which date back to around 2600 BC (Cragg and Newman, 2005).

People living in resource-poor communities in Nigeria and elsewhere continue to depend largely on natural product-derived medicines (particularly those of plant origin) to combat many pathological conditions, notwithstanding the dearth of pharmacological elucidation of their mechanisms of action and of standard clinical trials. This is often due to personal beliefs, economic reasons, or difficulty in accessing pharmaceutical products. In contrast to combinatorial chemistry, natural products provide enormous structural diversity, creating the opportunity to discover novel lead compounds. It is estimated that about 75,000 species of flowering plants are known to exist on earth, out of which only about 10% have been investigated for possible therapeutic value against any condition. Out of this 10%, only 1–5% have been scientifically researched for any bioactivity (Amit Koparde et al., 2019). Thus, countless important natural lead compounds, with a vast range of structures and pharmacological properties, await discovery in the Earth's biodiversity.

Many protozoan diseases are endemic in tropical countries, affecting millions of humans and animals, and causing serious economic losses annually, especially to developing economies. Most of these parasitic diseases attract little attention with regard to the development of new drugs, mainly because of poor profitability prospects. In addition, the development of resistance by these parasites to nearly all currently used chemotherapies, in addition to toxicity issues and the increasing cost of unrelated drugs, has led to an ever-increasing need to explore cheaper, accessible sources of safe new antiprotozoal agents (de Koning, 2017).

To date, a large number of Nigerian medicinal plant extracts has been successfully tested and found to show antiprotozoal activity (Lifongo et al., 2014). Several studies have reported remarkable *in vitro* and *in vivo* activity of extracts and fractions of Nigerian plants against *Plasmodium* spp. (Adebayo and Krettli, 2011), *Trypanosoma* spp. (Abiodun et al., 2012; Nwodo et al., 2015a), and *Leishmania* spp. (Bello et al., 2017). Indeed, promising lead compounds have been identified from some of these plants (Amoa Onguéné et al., 2013; Ntie-Kang et al., 2014; Bekono et al., 2020). However, due to research and resource limitations, the active principles of too many extracts from Nigerian medicinal plants remain unknown, and further studies that would facilitate the translation of the few identified compounds into drug candidates are limited by a lack of funding for such work (Ebiloma et al., 2018b). It should be noted that previous reviews have provided a compilation of evidence of antiprotozoal activity in materials collected from Nigerian flora. Other works have also documented bioactive compounds from plants that grow in Nigeria and the wider African continent, but not necessarily isolated from materials collected in the country or the region (Adebayo and Krettli, 2011; Nwodo et al., 2015a). With detailed analytical chemistry and bioactivity screening of plant materials collected specifically from Nigeria increasing, it has become opportune to document the compounds isolated from these plants, and their utility for development as antiprotozoal drugs. This would provide a guide for a focused and evidence-based approach for advancement and development of natural compounds from Nigeria and hence, from Africa in general.

In this review, we present an overview of some of the most important antimalarial, antitrypanosomal, and/or antileishmanial compounds that have been isolated from medicinal plant materials collected in Nigeria. An extensive internet-based search was carried out on PubMed, Web of Science, Science Direct, and Google Scholar using appropriate combinations of the keywords “antimalarial, antitrypanosomal, antileishmanial, plant, natural product, extract, compound, and Nigeria.” The last search was carried out on October 10, 2020 and only compounds with elucidated structures and reported half maximal inhibitory concentration (IC_50_), half maximal effective concentration (EC_50_) or minimum inhibitory concentration (MIC) of ≤50 μg/mL or μM were included. References lists of included and related studies were also screened visually to identify further relevant studies. Beyond evaluating the antiprotozoal utility of these natural drugs, the aim is to identify the current limitations in natural product drug discovery in Nigeria and elsewhere, recommend a way forward, and encourage further studies that would identify the actual translation of the best leads into new antiprotozoal chemotherapies, which are sorely needed.

## Plants as a Source of Antiparasitic Drugs

The annual global drug market is worth about one trillion USD (Calixto, 2019). A comprehensive analysis of drugs approved by the United States' Food and Drug Administration (FDA) from 1981 to 2010 shows that ~35% of these drugs were directly or indirectly of natural products origin, including plants (25% of total new pharmaceuticals) (Newman and Cragg, 2012, 2016). Over the last two centuries, the global pharmaceutical industry has significantly profited from the biodiversity of several countries when it comes to identifying new therapeutics against important pathogens, especially the development of new chemotypes for managing protozoal diseases such as malaria and leishmaniasis (Calixto, 2019).

Some of the natural compounds that have been isolated from plants include alkaloids, phenolics, terpenes, saponins, and quinones, and their derivatives (Table 1). They are valuable in combating diseases because of their anti-parasitic efficacies and their selective mode of action (Ebiloma et al., 2017). In addition to the direct usage of natural products from plants (phytomedicines or pure drugs), the scaffolds of many of these compounds have been successfully modified to produce pharmacologically more complex or semi-synthetic active molecules. They can also be used as taxonomic indicators for the discovery of new drugs; others can be used as models for designing lead molecules. Antique wisdom is the basis of modern medicine and a significant base of future medicines and therapeutics. Examples of well-established plant-based anti-protozoal drugs currently in use today are quinine and artemisinin, which are used for the treatment of malaria.

**Table 1:** Compounds isolated from Nigerian medicinal plants with antimalarial, antitrypanosomal, and antileishmanial activity.

**Compound**	**Compound class/ subclass**	**Part of plant studied**	**Species name**	**Plant family**	**Place of collection**	**Ethnomedicinal use of the plant**	**Activity of the compound**	**References**
**1**–**6**	Indole alkaloid	Fruits	*Picralima nitida*	Apocynaceae	Nnewi	Fever, jaundice, gastrointestinal disorders, and malaria	*Antiplasmodial*	Okunji et al., 2005
**7**	Benzophenanthridine alkaloid	Root	*Fagara zanthoxyloides*	Rutaceae	Ile-Ife, Osun State	Chewing sticks	*Antiplasmodial*	Kassim et al., 2005
**8**	Beta carboline Alkaloid	Bark	*Nauclea pobeguinii*	Rubiaceae	Oju LGA Benue State	Fever, jaundice, malaria, diarrhea	*Antitrypanosomal*	Igoli et al., 2011
**9** and **10**	Alkaloid	Seed	*Monodora myristica*	Annonaceae	Oju LGA Benue State	Fever, sepsis, stomach-ache, and constipation	*Antitrypanosomal*	Igoli et al., 2011
**11**–**14**	Isoquinoline alkaloid	Stembark	*Enantia chlorantha* Oliv.	Annonaceae	Ore, Ondo state	Stomach problems, rickettsia, typhoid fever, jaundice, malaria, and tuberculosis, some	*Antiplasmodial* and *antitrypanosomal*	Imieje et al., 2017
**15**–**24**	Steroid alkaloid	Leaves and stembark	*Holarrhena africana* (syn. *Holarrhena floribunda*)	Apocynaceae	Nsukka LGA Enugu State	Convulsion, Fever, malaria, snake venom antidote	*Antitrypanosomal*	Nnadi et al., 2017, 2019
**25**	Diterpenoid	Leaves	*Hyptis suaveolens*	Lamiaceae	South-Eastern Nigeria	Respiratory tract infections, colds, pain, fever, cramps, and skin diseases	Antiplasmodial	Chukwujekwu et al., 2005
**26**–**28**	Diterpenoid	Stembark	*Jatropha multifida*	Euphorbiaceae	Edo State	Hepatitis and leishmaniasis	Antiplasmodial and antileishmanial	Falodun et al., 2014
**29–32**	Diterpenes/ sesquiterpenes	Rhizomes	*Siphonochilus aethiopicus*	Zingiberaceae	Makurdi, Benue State	Colds, coughs, hysteria, infections, wound dressing, fevers, and pain	Antitrypanosomal	Igoli N. et al., 2011
**33**–**35**	Diterpenoid	Leaves	*Polyalthia longifolia*	Annonaceae	Anyigba, Kogi State	In the treatment of trypanosomiasis, leishmaniasis, and malaria	Trypanosomiasis	Ebiloma et al., 2017
**36**	Diterpenoid	Roots	Jatropha gossypiifolia	Euphorbiaceae	Benin City, Edo State	Leprosy, as an antidote for snakebite and in urinary problems	Antiplasmodial, antileishmanial	Ogbonna et al., 2017
**37**	Sesquiterpenes	Leaves	*Piliostigma thonningii*	Leguminosae	Abuja	Treatment of ulcers, wounds, heart pain, arthritis, malaria, fever, leprosy, sore throat, diarrhea, toothache, and cough	Antitrypanosomal	Afolayan et al., 2018
**38** and **39**	Labdane diterpenes	Leaves	*Piliostigma thonningii*	Leguminosae	Abuja	Treatment of ulcers, wounds, heart pain, arthritis, malaria, fever, leprosy, sore throat, diarrhea, toothache, and cough	Antitrypanosomal and antileishmanial	Afolayan et al., 2018
**40**	Cassane diterpene	Roots	*Calliandra portoricensis*	Fabaceae	Jos, Plateau State	Treatment of tuberculosis, constipation, and helminthiasis	Antitrypanosomal and antileishmanial	Nvau et al., 2020
**41**	Terpenoid/ diterpenoid	Leaves	*Eucalyptus maculata*	Myrtaceae	Anyigba, Kogi State	In the treatment of trypanosomiasis, leishmaniasis, and malaria	Antitrypanosomal	Ebiloma et al., 2017
**42**–**44**	Triterpenoid	stem bark	*Spathodea campanulata*	Bignoniaciaciae	–	Malaria	Antiplasmodial	Amusan et al., 1996
**45** and **46**	Triterpenoid	stem bark	*Khaya grandifoliola*	Meliaceae	–	Malaria and other febrile conditions	Antiplasmodial	Agbedahunsi and Elujoba, 1998
**47**	Triterpenoid		*Cassia siamea* L.	Fabaceae	Otu, Oyo State	As laxative and in treatment of insomnia, diabetes, and hypertension	Antiplasmodial	Ajaiyeoba et al., 2008
**48**	Triterpene	Leaves	*Combretum racemosum*	Combretaceae	Ibadan, Oyo State	Parasitic diseases and fever	Antiplasmodial	Oluyemi et al., 2020
**49**	Tetraterpenoids/ carotenoids	Leaves	*Bridelia ferruginea*	Euphorbiaceae	Eruwa, Oyo State	Purgative and vermifuge	Antitrypanosomal	Afolayan et al., 2019
**50**	Flavonol	Roots	*Spondias mombim*	Anacardiaceae	Oju LGA Benue State	Cough, sore throat, antiseptic soap, and malaria	Antitrypanosomal	Igoli et al., 2011
**51**	Flavonol	Bark	*Alcornea cordifolia*	Euphorbiaceae	Oju LGA Benue State	Fever, rheumatic pains, urinary tract infections, ringworm, and dysentery	Antitrypanosomal	Igoli et al., 2011
**52**	Flavonoid	Leaves	*Chromolaena odorata*	*Asteraceae*	Lagos State	Malaria	Antiplasmodial	Ezenyi et al., 2014
**53** and **54**	Flavonoid	Leaves	*Vitex simplicifolia*	Verbenaceae	Nsukka LGA, Enugu State	To treat edema, gout, malaria, skin diseases, toothache, and dermatitis	Antitrypanosomal	Nwodo et al., 2015a
**55**	Chalcone	Leaves	*Cajanus cajan*	Fabaceae	Otu, Oyo State	Laxative, antimalarial	Antiplasmodial	Ajaiyeoba et al., 2013
**56**	Anthraquinone	Leaves	*Mitracarpus scaber*	Rubiaceae	Zaria, Kaduna State	Headaches, skin and venereal diseases, toothaches, leprosy, amenorrhoea	Antitrypanosomal	Nok, 2002
**57**	Quinone	Whole plant	*Cassia nigricans*	Caesalpinaceae	Kaduna State	Stomach ulcers	Antiplasmodial	Obodozie et al., 2004
**57**	Quinone	Stem bark	*Cassia siamea*	Fabaceae	Otu in Oyo State	For insomnia, as laxative and antidiabetic	Antiplasmodial	Ajaiyeoba et al., 2008
**58**	Anthraquinone	Leaves	*Crateva adansonii*	Capparaceae	Nsukka, Enugu State	Treatment of headaches, ear, and parasitic infections	*Antitrypanosomal*	Igoli et al., 2014
**59** and **60**	Isoflavanquinones	Roots	*Abrus precatorius*	Fabaceae	Umuoriehi Umuahia, Abia State	Diabetes, hemoglobinuria, sore throat, rheumatism, skin infections, and jaundice	Antileishmanial	Okoro et al., 2020
**61**–**65**	Tannins	stem bark	*Terminalia avicennoides* and *Anogeissus leiocarpus*	Combretaceae	Bauchi State	TA: cure dental carries and skin infections AL: diarrhea, dysentery, and malaria,	Antiplasmodial, antitrypanosomal and antileishmanial	Shuaibu et al., 2008a,b,c
**66**	Coumarin	Stem	*Euphorbia poisonii*	Euphorbiaceae	Oju LGA Benue State	Pain killer, laxative, pesticide, arrow-poison	Antitrypanosomal	Igoli et al., 2011
**67**	Saponins	Seed	*Dracaena mannii* (DM) and *D. arborea* (DA)	Dracaenaceae	Isi-Enu, Nsukka, Enugu State	DM: Stomach-ache, mental illness gonorrhea, and chest pains DA: epilepsy, measles, smallpox, venereal diseases	Antiplasmodial	Okunji et al., 1996
**68**–**70**	Glycoside/glucoside	Leaves	Stachytarpheta cayennensis	Verbenaceae	–	Malaria, helminthiasis fever and constipation	Antiplasmodial	Ifeoma Chinwude et al., 2016
**71** and **72**	Irodid glucoside	Leaves	*Vitex grandifolia*	Lamiaceae	Ilorin, Kwara State	Colic, umbilical cord infections, toothache, rheumatism, and orchitis	Antileishmaial	Bello et al., 2018
**73**	Peptide	Leaves	*Crateva adansonii* DC		Nsukka, Enugu State	Treatment of headaches, ear-aches, and parasitic infections	*Antitrypanosomal*	Igoli et al., 2014
**74** and **75**	Amine (dipeptides)	Roots	*Zapoteca portoricensis*	Fabaceae	Nsukka, Enugu State	For wound healing, toothache, tonsilitis, diarrhea, and as an anticonvulsant and antispasmodic	*Antitrypanosomal*	Nwodo et al., 2014
**76** and **77**	Fatty acids	Leaves	*Carica papaya*	Caricaceae	Abia State	Asthma, rheumatism, fever, diarrhea, boils, and hypertension and as lactogenic	Antiplasmodial	Melariri et al., 2011
**78** and **79**	Taccalonolide	Tubers	*Tacca leontopetaloides*	Taccaceae	Benue State	Stomach disorders, gastric ulcers, tooth ache, high blood pressure, hepatitis, enteritis, and sexual dysfunction	*Antitrypanosomal*	Dike et al., 2016
**80** and **81**	Pheophorbide	Leaves	*Crateva adansonii* DC	Capparaceae	Nsukka, Enugu State	Treatment of headaches, ear, and parasitic infections.	*Antitrypanosomal*	Igoli et al., 2014
**82** and **83**	Pheophytins	Leaves	*Newbouldia laevis*	*Bignoniaceae*	Anyigba, Kogi State	To hasten parturition and placenta delivery	Antitrypanosomal	Ebiloma et al., 2017

For centuries, the bark of *Cinchona* species was used by the natives living in the Amazon area for the treatment of malaria caused by *Plasmodium*. Interestingly, as early as 1820, Pelletier and Caventou successfully isolated quinine, the bioactive principle, from the bark of *Cinchona officinalis* (Dias et al., 2012). Consequently, the curative agent for malaria in those early days was quinine. Subsequently, several synthetic derivatives including chloroquine, mefloquine, amodiaquine, and primaquine were developed. The long history of usage notwithstanding, i.e., first as extract preparations and later as pure compound, malaria chemotherapy is still to an extent dependent on quinine; this is especially true for the most lethal form, cerebral malaria. Interestingly, while resistance to the synthetic derivatives such as chloroquine developed rapidly, resistance to quinine itself is relatively rare (Achan et al., 2011; Okombo et al., 2011).

The remarkable contributions of plants to the discovery of antiprotozoal drugs would not be complete without mentioning the plant *Artemisia annua. A. annua* is another plant that was long used for the treatment of fevers, in this case in Chinese folkloric medicine. The successful isolation of the bioactive agent, Artemether [a strong anti-malarial agent that was derived from artemisinin (qinghaosu), a sesquiterpene lactone], from *A. annua* represents one of the most significant discoveries in the fight against diseases caused by a protozoan parasite (Tu, 2011). Since then, several semi-synthetic artemisinin-based derivatives have been approved and registered for use: artesunate, dihydroartemisinin, and artemether (Paddon and Keasling, 2014).

## Active Compounds From Nigerian Plants

### Alkaloids

**Figure 1:**
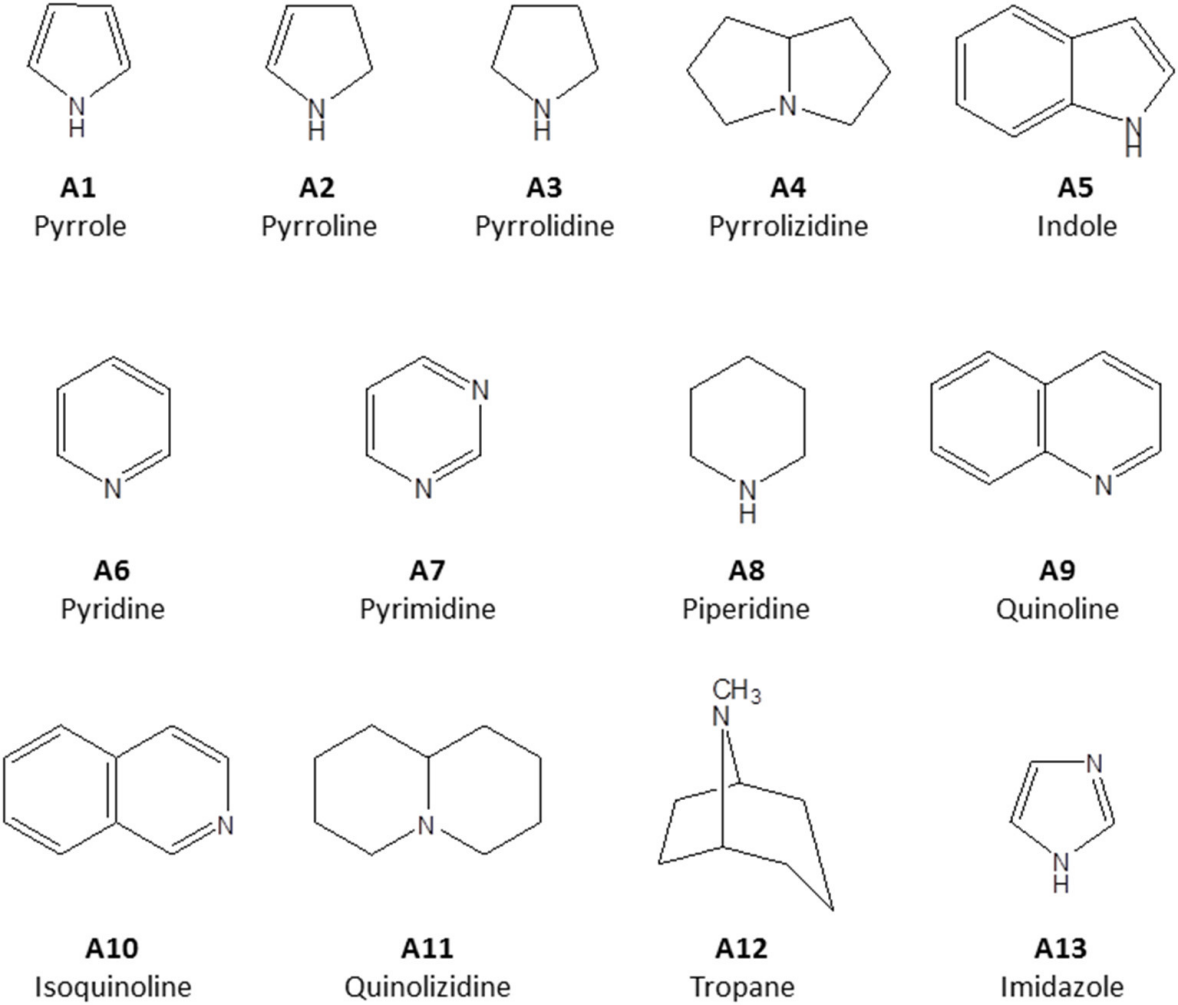
Structure of principal C-N skeleton of alkaloid sub-groups.

Alkaloids are a structurally diverse class of secondary metabolites whose key feature is a basic nitrogen (nitrogen in a negative oxidation state) in a carbon ring. They are classified according to their principal C-N skeleton into pyrroles (A1), pyrrolines (A2), pyrrolidines (A3), pyrrolizidines (A4) indoles (A5), pyridines (A6), pyrimidines (A7), piperidines (A8) quinolines (A9), isoquinolines (A10), quinolizidines (A11), tropanes (A12), imidazoles (A13) (Figure 1). They can also be classified according to their biological origin. Alkaloids are widely distributed in plants and some animal species. For many years, plant alkaloids have been utilized in the treatment of various health conditions (Bribi, 2018).

**Figure 2:**
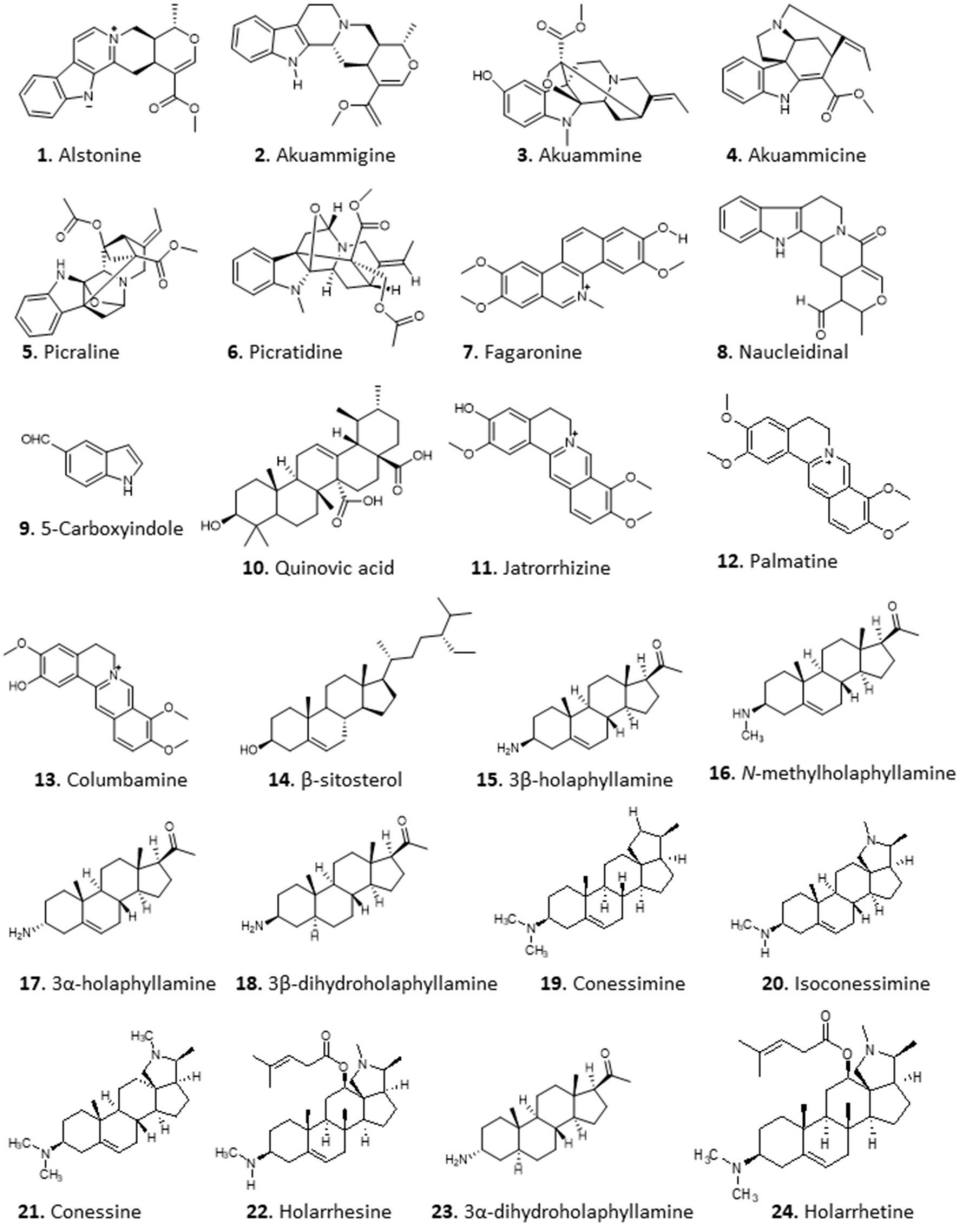
Structure of alkaloids isolated from Nigerian plants with selected antiprotozoal activity.

An excellent example is the isolation of anti-malarial alkaloids from the fruit rind of *Picralima nitida*. The crude dichloromethane extract displayed *in vitro* antiplasmodial activity with half maximal inhibitory concentration (IC_50_) values of 1.6–2.4 μg/mL. Moreover, the methanolic extract of the plant's stem bark, root, and fruit rind yielded active alkaloid fractions with IC_50_s of 0.54–2.16 and 0.79–1.59 μg/mL against *P. falciparum* chloroquine-resistant (W2) and chloroquine-sensitive (D6) clones, respectively (Iwu and Kiayman, 1992). Using pH-zone-refining counter-current chromatography, Okunji et al. (2005) isolated indole alkaloids from the rind of the plant. The purified compounds, alstonine (**1**), akuammigine (**2**), akuammine (**3**), akuammicine (**4**), picraline (**5**), and picratidine (**6**), showed remarkable activity against both *P. falciparum* clones (D6 and W2; IC_50_ 0.01–0.9 μg/mL) (Okunji et al., 2005) (for the structures of compounds **1**–**24** see Figure 2).

The crude, semi-purified and purified aqueous extracts of the roots of *Fagara zanthoxyloides*, known as Senegal prickly-ash, displayed highly promising antiplasmodial activity with IC_50_ values of 4.90, 1.00, and 0.13 μg/mL, respectively, using the [^3^H]-hypoxanthine incorporation assay. Further separation yielded a very potent antiplasmodial benzophenanthridine alkaloid, fagaronine (**7**) with an IC_50_ of 0.018 μg/mL against *P. falciparum* strain 3D7 (Kassim et al., 2005). A short and efficient synthesis for this compound was developed by Rivaud et al. (2012), who also confirmed the compound's activity against chloroquine-sensitive and -resistant *P. falciparum* isolates superior to chloroquine with half maximal effective concentration (EC_50_) of ~10 nM *in vitro* and comparable to chloroquine against *P. vinckei in vivo* [median effective dose (ED_50_) of 6 mg/Kg/day for 4 days]. Moreover, the compound, displayed no toxicity against Vero cells *in vitro* (Rivaud et al., 2012) or by single injection in the mouse at up to 50 mg/Kg (Nakanishi et al., 1999). The methanol extract of *F. zanthoxyloides* also displayed >90% inhibition of *Plasmodium berghei in vivo*, with an estimated EC_50_ of 235 mg/Kg b.w. for the extract while the lethal dose 50 (LD_50_) was >5,000 mg/Kg b.w. in mice (Enechi et al., 2019), providing independent confirmation of the antimalarial potential of this plant.

A preparation of 80% ethanolic extract of the stem bark of *Nauclea pobeguinii*, containing 5.6% of the beta-carboline alkaloid strictosamide as the presumed active agent, displayed highly promising activity against *falciparum* malaria in a Phase IIB clinical trial as a herbal medicine (Mesia et al., 2012). A beta carboline alkaloid, **8**, was isolated from the ethyl acetate extract of the bark of the same plant and showed moderate activity against the causative agent of sleeping sickness, *Trypanosoma brucei*, with a Minimum Inhibitory Concentration (MIC) of 12.5 μg/mL and low cytotoxicity against PNT2A cells with IC_50_ >100 μg/mL (Igoli et al., 2011). Two other alkaloids, compounds **9** and **10**, with similar moderate trypanocidal activity were obtained from the ethyl acetate extract of *Monodora myristica*, showing MICs of 12.5 and 25 μg/mL, respectively, for *T. brucei* (Igoli et al., 2011).

Imieje et al. (2017) screened the crude methanolic extract and different fractions of *Enantia chlorantha* (African Whitewood, family Annonaceae), which is used in Nigerian ethnomedicine for the treatment of wounds, ulcers, and fevers including malaria, for antiprotozoal activity and reported that the extract indeed displayed very high activity against *P. falciparum*, with very good IC_50_ values against chloroquine-sensitive D6 (<0.37 μg/mL) and chloroquine-resistant W2 (<0.32 μg/mL) clones as well as against *Leishmania donovani* (IC_50_ <0.8 μg/mL), but only moderate activity against *T. brucei* (IC_50_ of 15.2 μg/mL). The butanol and ethanol fractions of the extract yielded three closely related isoquinoline alkaloids: jatrorrhizine (**11**), palmatine (**12**), and columbamine (**13**). The steroid β-sitosterol (**14**) was also obtained from the extracts. Compound **11** displayed good but only moderately specific activity against chloroquine-sensitive *P. falciparum* clone D6 (IC_50_ 2.2 μM), but activity against *L. donovani* and *T. brucei* was low (IC_50_s >29.6 and 18.3 μM, respectively), and IC_50_s >14.1 and >29.6 to VERO and human acute monocytic leukemia (THP1) cells, respectively. Compounds **12** and **13** showed essentially identical activity as **11** against *P. falciparum* D6 with IC_50_ of 1.90 and 2.16 μM, respectively, showing that the methylation of the isoquinoline hydroxy groups is not essential for activity, whereas **14** displayed only moderate activity (Pf IC_50_ >11.5). Compounds **12** and **14** were also tested against *L. donovani* and *T. brucei*, with IC_50_ values ≥23.1 μM (Imieje et al., 2017).

The *in vitro* and *in vivo* antitrypanosomal activity of the aqueous extract of young leaves of *Holarrhena africana* from Nigeria has been reported by multiple authors (Nwodo et al., 2007; Alhaji et al., 2014; Nnadi et al., 2016). Nnadi et al. investigated the antitrypanosomal, antileishmanial and antiplasmodial activity of this plant in more detail, using extracts and fractions from both stem bark and leaves (Nnadi et al., 2016, 2017). The crude extract and alkaloid fraction both displayed potent antitrypanosomal activity. Bioactivity guided fractionation of the active alkaloid fraction led to the isolation of 19 compounds, 17 of which were steroid alkaloids. Remarkable activities (EC_50_ in μM ± absolute deviation) against *T. brucei* rhodesiense were recorded for some aminosteroids from the leaves: 3β-holaphyllamine (0.40 ± 0.28) **15**, N-methylholaphyllamine (0.08 ± 0.01) **16**, 3α-holaphyllamine (0.37 ± 0.16) **17** and 3β-dihydroholaphyllamine (0.67 ± 0.03) **18**. Related compounds from the stembark, Conessimine (0.17 ± 0.08) **19**; isoconessimine (0.17 ± 0.11) **20**; conessine (0.42 ± 0.09) **21**; and holarrhesine (0.12 ± 0.08) **22** also showed very high antitrypanosomal activity, with high selectivity to the parasites [selectivity index (SI) >100] compared to mammalian L6 cells (Nnadi et al., 2017). A 3D-Quantitave Structure Activity Relationship (QSAR) study revealed that steric activity around the C-3 amino group tends to increase activity, while steric activity in the vicinity of the amino group of the pyrroline/pyrrolidine rings and the C-17β-acetyl or C-20 methyl groups tended to decrease activity (Nnadi et al., 2018). Following on from this, the active aminosteroids together with **22**, 3α- dihydroholaphyllamine (**23**), and holarrhetine (**24**) were also tested on animal trypanosome species using resazurin-based *in vitro* screening assay in our lab (Nnadi et al., 2019). Interestingly, most of these compounds displayed no cross-resistance with pentamidine, isometamidium, diamidines, and melaminophenyl arsenicals in *T. b. brucei*. The activity of these compounds varies between *T. b. brucei* and *T. congolense*, suggesting possible differences in the mode of action of these compounds in the 2 species as seen with some trypanocides. The compounds with highest activity (IC_50_ ± standard deviation) and selectivity [represented as Selectivity Index (SI)] against *T. congolense* were **20** (IC_50_ = 0.22 ± 0.35 μM; SI = 123.5), **21** (IC_50_ = 1.65 ± 0.92 μM; SI = 37.2), **22** (IC_50_ = 0.22 ± 0.13 μM; SI = 65.9), and **23** (IC_50_ = 0.045 ± 0.03 μM; SI = 2,130) (Nnadi et al., 2019). Further exploration into the mechanism of action revealed that the compounds caused a slow but irreversible trypanocidal effect by targeting the mitochondrion, preventing kinetoplast division (Nnadi et al., 2019).

### Terpenoids

**Figure 3:**
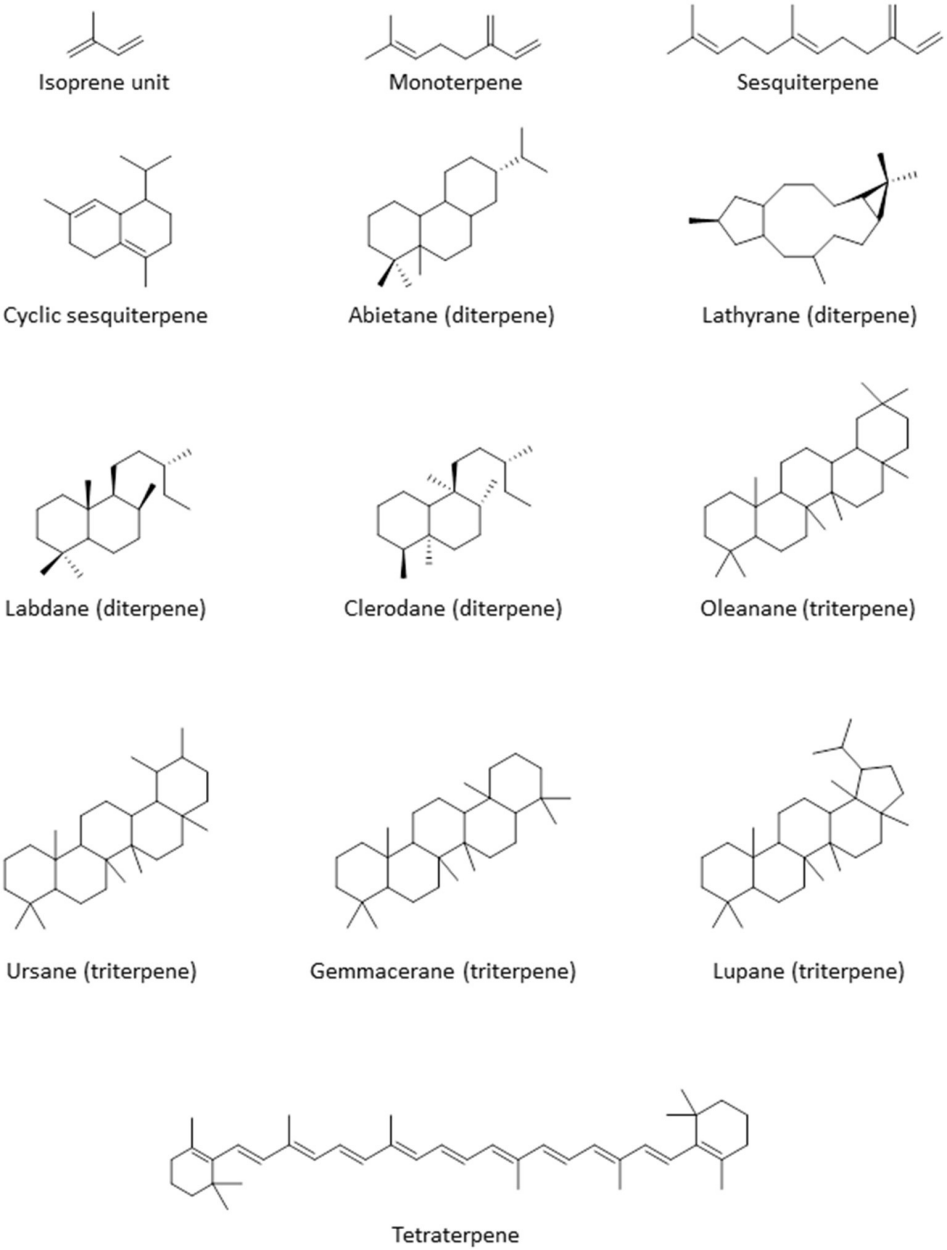
Structure of the basic skeleton of terpene sub-groups.

Terpenoids are commonly found in plants and are biosynthesized from isoprene units (CH_2_=C(CH_3_)-CH=CH_2_). They are classified based on the number of units in the compound; monoterpenes consist of 2 units, while sesquiterpenes, diterpenes, triterpenes, and tetraterpenes consist of 3, 4, 6, and 8 units, respectively (Figure 3). The number of carbon atoms in the compounds are thus 10, 15, 20, 30, and 40 depending on the number of isoprene units. Terpenoids have shown good activity in various assay screens and constitute the active entities in many drugs. Compound **25** (9α, 13α-epi-dioxiabiet-8(14)-en-18-ol) was obtained through a bioassay-guided fractionation of the petroleum ether extract of the leaves of Hyptis suaveolens, a common shrub in the tropics, used traditionally for the treatment of many ailments including fever, colds, and respiratory tract infections (Iwu, 2014). The compound demonstrated high antiplasmodial activity with an IC_50_ of 0.1 μg/mL against chloroquine-sensitive *P. falciparum* clone D10 (Chukwujekwu et al., 2005).

The methanol extract of Jatropha multifidi, known as coral bush, which is in fact used in Nigerian folk medicine for the treatment of parasitic infections as well as cancer (Gill, 1992), yielded 3 closely related macrocyclic lathyrane diterpenoids: 14-deoxy-1β-hydroxy-4(4E)-jatrogrossidentadione **(26)**, the saturated 15-deoxy-1β-hydroxy-4(4E)-jatrogrossidentadione **(27)** and the unsaturated 15-deoxy-1β-hydroxy-4(4E)-jatrogrossidentadione **(28)** (Falodun et al., 2014). The compounds showed particular promise for their antileishmanial activity, with IC_50_ of 11.9, 4.69, and 4.56 μg/mL, respectively, showing that the oxidation state of the oxygen on the cyclopentene ring is likely to be important for high activity. The authors state that traditional healers have used the plant successfully against leishmaniasis. The compounds possessed virtually no activity against chloroquine-sensitive *P. falciparum* D6, with IC_50_s >7 mg/mL, and poor activity against a panel of bacterial and fungal pathogens, thus confirming specific activity against *Leishmania* species and perhaps other kinetoplastid parasites (Falodun et al., 2014).

The crude hexane extracts of the rhizomes of wild ginger, *Siphonochilus aethiopicus*, showed promising activity against *T. brucei* with an MIC of 6.5 μg/mL and low toxicity against several mammalian cells (MIC of 53.3–114.2 μg/mL). Two active diterpenes, 8(17),12E-labdadiene15,16-dial **(29)**, 15-hydroxy-8(17),12E-labdadiene-16-al **(30)** and two sesquiterpenes, epi-curzerenone **(31)**, furanodienone **(32)** were isolated from the extract (Igoli N. et al., 2011). **29, 31, 32** showed a MIC of 1.55 μg/mL while **30** displayed an MIC of 6.25 μg/mL against *T. brucei*. The compounds appeared to be selective for *T. brucei* as cytotoxicity tests with the mammalian cells showed MICs of 35.9–116 μg/mL, except for **29**, which was toxic to Jurkat and SH-SY5Y cells (MIC 4.1 and 9.0 μg/mL, respectively) (Igoli N. et al., 2011).

The Indian-native ornamental plant *Polyalthia longifolia*, commonly found in Nigeria is an important source of traditional remedies for malaria and fevers (Bankole et al., 2016). The hexane extract of the leaves of *P. longifolia* showed potent activity against *T. b. brucei* in our lab, with EC_50_ ± SEM of 2.4 ± 0.1 μg/mL. Bioactivity-guided fractionation led to the isolation of a diterpenoid identified as clerodane (16-α-hyroxy-cleroda-3-13(-14)-Z-dien-15,16-olide; compound **33**), which displayed an EC_50_ ± SEM of 0.38 ± 0.05 μg/mL against *T. b. brucei*. Other isolated compounds from the fraction were polyalthialdioc acid **(34)** and kolavenic acid **(35)**, which also showed significant antitrypanosomal activity, with EC_50_ ± SEM values of 3.57 ± 0.16 and 12.3 ± 0.5 μg/mL, respectively. Interestingly, all the 3 compounds also showed activity against *T. congolense* and *L. mexicana*, presented no cross-resistance to diamidines and arsenicals, and were not toxic to Human Embryonic Kidney (HEK) cells at up to 200 μg/mL (Ebiloma et al., 2017). Compound **33** has also been previously described as an oral antileishmanial agent with *in vivo* activity (Misra et al., 2010). We further investigated the effect of **33** and reported a multi-target mechanism of action for the compound on *T. brucei*, including severe cell cycle defects, DNA fragmentation, ATP depletion, and a marked depolarisation of the mitochondrial membrane potential (Ebiloma et al., 2018b).

Chromatographic fractionation and separation of fractions of the methanol extract of the root bark of *Jatropha gossypifolia* (“bellyache bush”), a pantropical plant with many ethnopharmacological applications for its various parts (Félix-Silva et al., 2014), yielded a macrocyclic diterpenoid compound, jatrophone **(36)**, with antiprotozoal activity. The compound displayed broad antiparasitic activity, with low EC_50_ values against chloroquine-sensitive *P. falciparum* clone D6 (0.55 μg/mL), chloroquine-resistant *P. falciparum* clone W2 (<0.52 μg/mL), *L. donovani* (<0.4 μg/mL), and *T. brucei* (<0.4 μg/mL). However, **36** was also found to be similarly active against VERO cells (0.43 μg/mL), and its antiprotozoal effects thus reflect a more general toxicity (Ogbonna et al., 2017).

Several compounds including a bioactive sesquiterpene, 2β-methoxyclovan-9α-ol **(37)**, two active labdane diterpenes, methyl-*ent*-3β-hydroxylabd-8(17)-en-15-oate **(38)** and alepterolic acid **(39)** were obtained from the methanol extract of the leaves of *Piliostigma thonningii*, a plant of the subfamily Caesalpinioideae in the legume family with myriad ethnopharmacological uses. Compounds **37** and **39** were antitrypanosomal with IC_50_s of 7.89 and 3.42 μM against *T. brucei*, respectively. Compound **38** displayed a broader antikinetoplastid activity with IC_50_ of 3.84 and 7.82 μM in *T. brucei* and *L. donovani*, respectively. The authors suggested that hydroxylation of the sesquiterpenes at C-2 position improves the antileishmanial activity, while hydroxylation at C-3 enhances the antitrypanosomal activity of the labdane diterpenes (Afolayan et al., 2018).

Column chromatography of the ethyl acetate extract of the roots of *Calliandra portericensis* yielded a novel diterpene-substituted chromanyl benzoquinone, bokkosin, **40**. The compound showed potent activity against the kinetoplastid parasites, *T. brucei* (0.69 μg/mL), *T. congolense* (21.6 μg/mL), and *L. mexicana* (5.8 μg/mL). In addition, it exhibited very low prospects of cross-resistance to antitrypanosomal drugs, pentamidine and diminazene, and low toxicity to mammalian cell lines (Nvau et al., 2020). The ethyl acetate extracts of the leaves of *Eucalyptus maculata* also exhibited antitrypanosomal activity (12.3 ± 0.3 μg/mL, EC_50_ ± SEM). An ursane type triterpenoid, 3β,13β-dihydroxy-urs-11-en-28-oic acid (41) isolated from this extract appeared to be the active ingredient, with EC_50_ ± SEM of 1.58 ± 0.03 μg/mL against *T. b. brucei*. Compound 41 showed no cross-resistance to diamidines and arsenicals, and was not toxic to HEK cells at concentrations up to 200 μg/mL (Ebiloma et al., 2017).

Amusan et al. (1996) reported the isolation of antimalarial triterpenoids from the chloroform extract of the stem bark of one of the plants that is tradionally used for the treatment of malaria, *Spathodea campanulata* (Makinde et al., 1987, 1988a). The triterpenoids, 3β-hydroxyurs-12-en-28-oic acid (ursolic acid; **42**) and two of its derivatives, 3β-hydroxyurs-12,19-dien-28-oic acid (tomentosolic acid; **43**) and 3β,20 β-dihydroxyurs-12-en-28-oic (**44**) resulted in a significant (*P* < 0.05) reduction in parasitaemia and enhanced survival of mice infected with *P. berghei*. Notably, the effect of 60 mg/Kg/day of **42** on parasitaemia and survival was comparable to 10 mg/Kg/day chloroquine (Amusan et al., 1996).

The same group reported that aqueous extracts of another Nigerian antimalarial plant, *Khaya grandifoliola* (African mahogany), also displayed activity against *P. berghei* infection in mice (Makinde et al., 1988b), but this extract only suppressed early infections and was ineffective against established infections of the parasite. However, Agbedahunsi et al. re-tested the antimalarial activity of *K. grandifoliola* stem bark extracts using different solvents (Agbedahunsi et al., 1998). Among the extracts tested, the n-hexane crude extract and purified fractions displayed the highest activities, comparable to the reference drug chloroquine diphosphate, with EC_50_ values of 1.4 μg/mL (for a multi-drug resistant clone) or 0.84 μg/mL (for Nigerian *P. falciparum* isolates). Further studies on the active n-hexane fraction yielded a tetranortriterpenoid, methyl-6-acetoxy angolensate (**45**), and a novel compound, grandifolin (**46**) (Agbedahunsi and Elujoba, 1998). Another triterpenoid, lupeol (**47**), isolated from methanol of *Cassia siamea* stem bark extract showed an IC_50_ of 5 μg/mL against *P. falciparum* using a parasite lactate dehydrogenase (pLDH) assay (Ajaiyeoba et al., 2008). Phytochemical investigation of the methanolic leaf extract of *Combretum racemosum* led to the identification of ursane-type triterpenes, with the most active antiplasmodial compound being madecassic acid (**48**) with mean EC_50_ ± SD values of 28 ± 12 and 17 ± 4 μg/mL against chloroquine-sensitive (D10) and chloroquine-resistant (W2) *P. falciparum*, respectively (Oluyemi et al., 2020).

**Figure 4:**
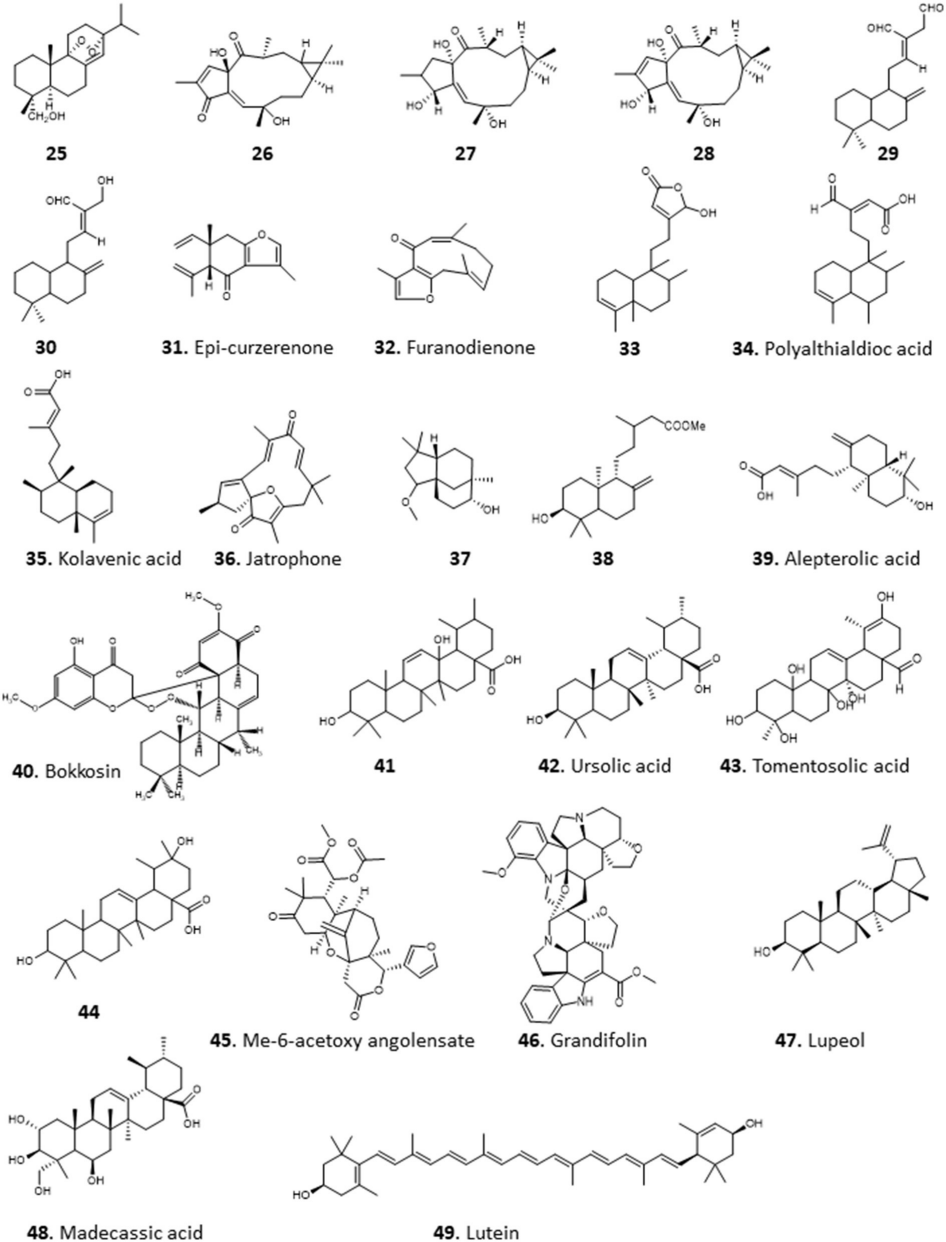
Structure of terpenoids isolated from Nigerian plants with selected antiprotozoal activity.

The crude methanolic extract of the leaves of *Bridelia ferruginea* displayed potent activity against *T. brucei* with >90% inhibition and EC_50_ of 8.48 μM. A tetraterpenoid/carotenoid, lutein (49), was isolated from the extract among other compound and showed activity against *T. brucei* (EC_50_ 4.16 μM) and *L. donovani* (EC_50_ 9.3 μM) (Afolayan et al., 2019) (for the structures of compounds **25**–**49**, see Figure 4).

### Flavonoids and Chalcones

**Figure 5:**
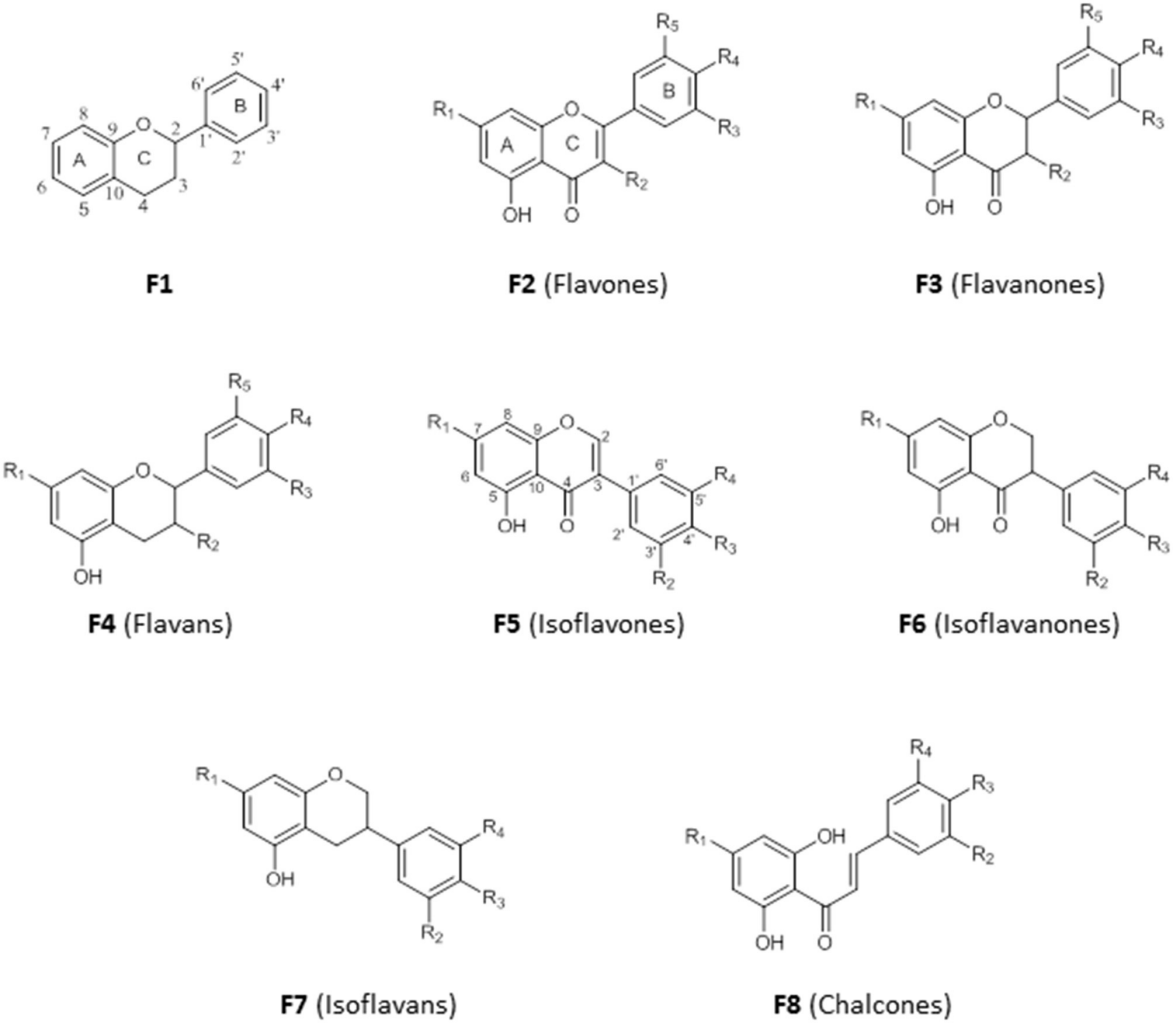
Structure of basic skeleton of flavonoid sub-groups.

Flavonoids are secondary metabolites with diverse medicinal properties and are commonly found in fruits, leaves, and flowers of plants. They are phenolics, with structures based on a 15-carbon skeleton made of two benzene rings (A and B) connected by a pyran ring (C) (F1). The saturation (F3) or unsaturation (F2) of the pyran ring, absence of the carbonyl group at C-4 (F4), connection of ring B to ring C at C-3 (F5, F6, F7) and the opening of ring C (F8) is the basis for their classification into flavones, flavanones, flavans, isoflavone, isoflavanones, isoflavans, and chalcones (Figure 5). An –OH substituent at C-3 on the flavones skeleton (F2) leads to flavonols, while on flavanone skeleton (F3) leads to flavanonols.

We have reported the isolation of two flavonols, **50** and **51**, from the combined hexane and ethyl acetate extracts of the roots of *Spondias mombim* and the methanolic extract of the bark of *Alcornea cordifolia*, respectively. Compound **50** displayed poor activity against *T. brucei* (EC_50_ 25 μg/mL) whereas **51**, different only by a single hydroxy substituent on the 5′ position, showed quite remarkable activity against this parasite (EC_50_ <0.2 μg/mL). However, **51** also displayed toxicity against PNT2A prostatic cells with an EC_50_ of 1.5 μg/mL and would thus be too toxic for therapeutic use (Igoli et al., 2011). Nonetheless, it might be worth exploring structural variations with the 5′-OH substitution in a SAR study.

The methanol extract of the leaves of the common tropical shrub *Chromolaena odorata*, extracts of which are used to treat malaria in South-Eastern Nigeria, suppressed parasitaemia in mice infected with *P. berghei* by 99.2 and 97.8% at 200 and 400 mg/Kg/day, respectively. The extract yielded a flavonoid identified as 3, 5, 7, 3′-tetrahydroxy-4′-methoxyflavone (**52**), which displayed high antiplasmodial activity, with 81.5% suppression of parasitaemia in mice infected with *P. berghei* at 2.5 mg/Kg/day, even better than the control chloroquine and artemether (Ezenyi et al., 2014). Nwodo et al. (2015b) isolated seven flavonoid derivatives from the leaves of *Vitex simplicifolia* (family Verbenaceae), which is used to treat trypanosomiasis in Nigeria, and evaluated their activity against *T. b. rhodesiense* as well as cytotoxicity in L6 cells. The most active and least toxic of the compounds were 2-(5′-methoxyphenyl)-3,4′,5,7,8-trihydroxychroman-4-one **(53)** with IC_50_ = 10.2 μg/mL and SI = 9.8, as well as artemetin **(54)** with IC_50_ = 4.7 μg/mL and SI = 9.8 (Nwodo et al., 2015b). This level of selectivity seems insufficient for any clinical development.

**Figure 6:**
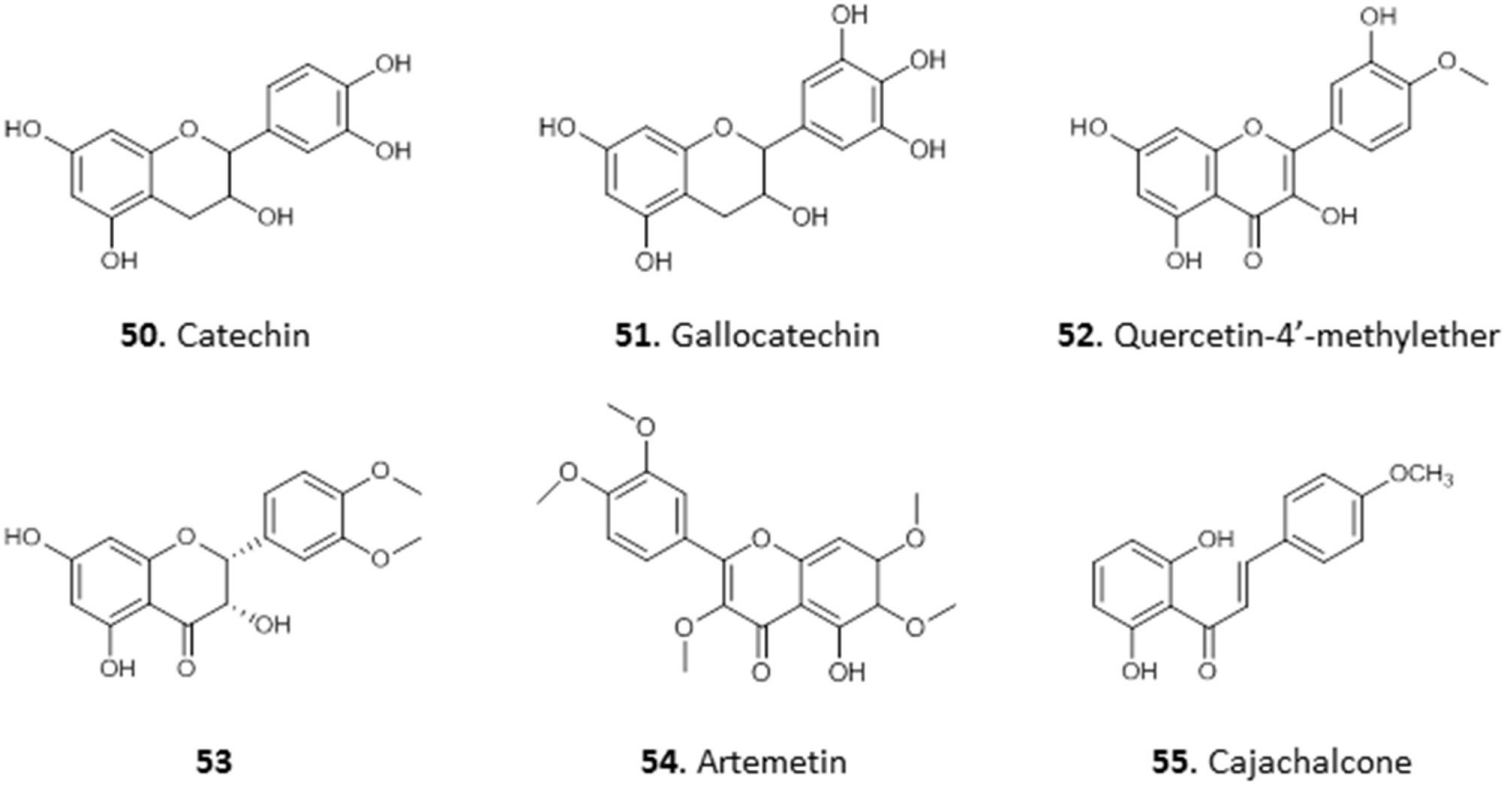
Structure of flavonoids isolated from Nigerian plants with selected antiprotozoal activity.

The crude methanol extract of the leaves of *Cajanus cajan*, a member of the family Fabaceae used for its antimalarial properties, nevertheless had an IC_50_ value of just 53.5 μg/mL against the multidrug-resistant *P. falciparum* K1 strain *in vitro*; however, its ethyl acetate fraction yielded cajachalcone, 2′,6′-dihydroxy-4-methoxy chalcone **(55)** with IC_50_ of 2.0 μg/mL (7.4 μM) (Ajaiyeoba et al., 2013). The relatively simple structures of these flavonoids and chalcones, which lack chiral centers, should enable a systematic exploration of their structure-activity relationships (for the structures of compounds **50**–**55**, see Figure 6).

### Quinones

**Figure 7:**
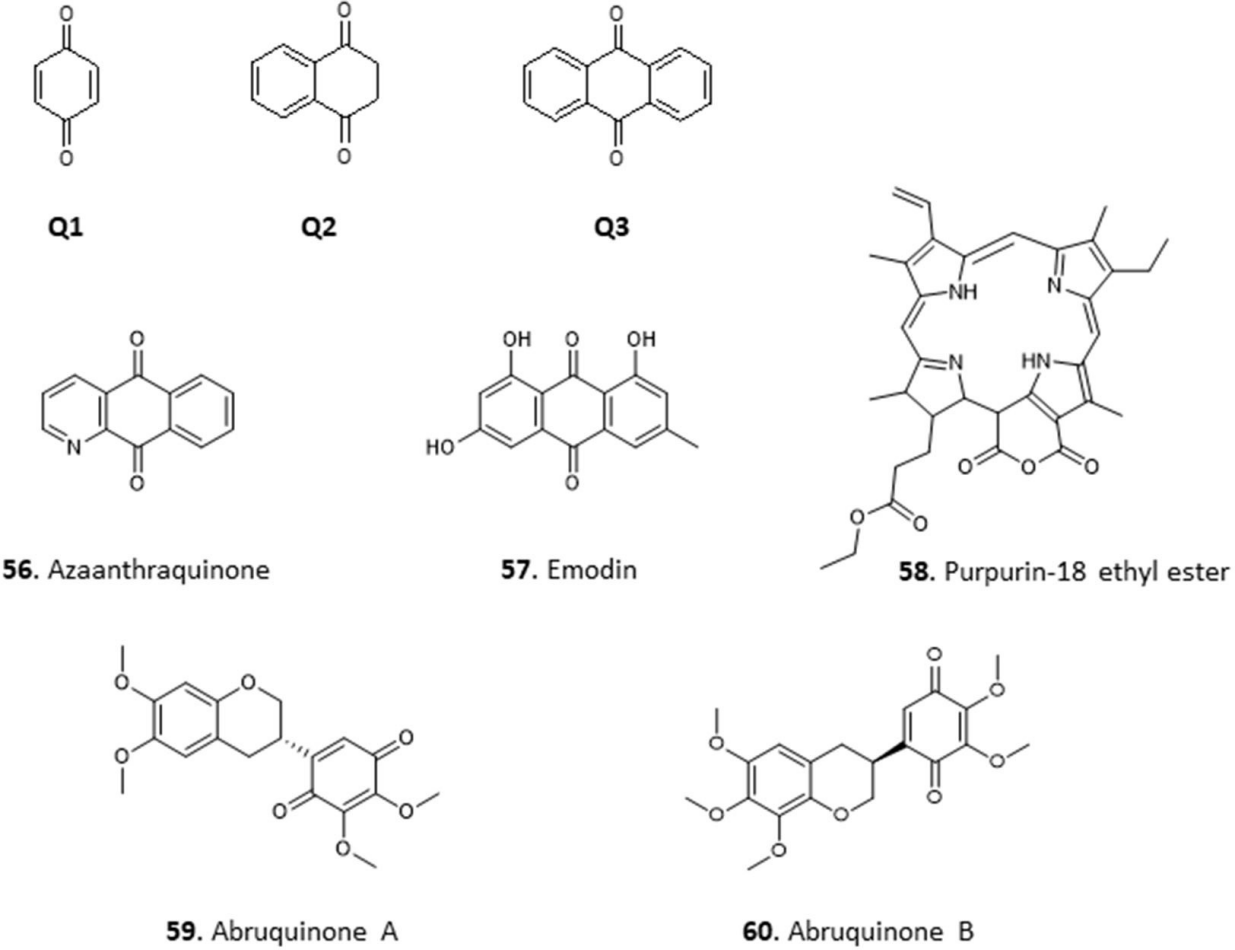
Structure of basic skeleton of quinone sub-groups (Q1–Q3) and quinones isolated from Nigerian plants with selected antiprotozoal activity (56–60).

The common feature among quinones is the presence of two carbonyl groups in a six-membered unsaturated ring (carbonyl groups can also be on adjacent rings). They are classified into three main groups: benzoquinones which have two carbonyl groups in a benzene ring (Q1), naphthoquinones, which have two carbonyl groups on one of the rings in naphthalene (Q2) and anthraquinones, which are derivatives of anthracene (Q3) (Figure 7). They are found in the leaves, seeds, and woody parts of higher plants, in some fungi and bacteria but rarely in higher animals. Quinones have been reported to have antioxidant, anti-inflammatory, antibiotic, antimicrobial, and anticancer activities (El-Najjar et al., 2011).

Treatment of *Trypanosoma congolense*-infected mice with the crude ethanol extract of the leaves of *Mitracarpus scaber*, a popular medicinal plant with known antifungal and antimicrobial properties (Irobi and Daramola, 1993; Ekpendu et al., 1994), at 50 and 150 mg/Kg/day cleared parasitaemia within 6 and 2 days, respectively, with no relapse for about 2 months. Benz(g)isoquinoline 5,10 dione (Azaanthraquinone; **56**) was isolated from the extract. It completely lysed *T. congolense* cells within 60 min at a concentration of 5 μM, and dose-dependently inhibited their motility and respiration (Nok, 2002). Nok investigated the mechanism of action further and concluded that this *M. scaber* quinone (**56**) interferes with the essential function of coenzyme Q (ubiquinone) in the trypanosomes, which carries electrons for aerobic respiration from mitochondrial glycerol-3-phosphate dehydrogenase to the Trypanosome Alternative Oxidase (TAO) (Ebiloma et al., 2018b). This is a rare example where the mechanism of the antiparasite action of a natural compound is well-understood. Considering that *T. brucei* spp. are even more susceptible to TAO inhibitors than *T. congolense* (Ebiloma et al., 2018a), due to differences in their mitochondrial pathways, it would be worth revisiting the wider trypanocidal activities of **56** (for the structures of compounds **56**–**60**, see Figure 7).

*Cassia nigricans* is a herbal plant used to treat various fevers in Nigeria. The methanol extract of the whole plant yielded an antiplasmodial compound, emodin **(57)**, an anthraquinone with an IC_50_ of 10.8 μg/mL in chloroquine-resistant *P. falciparum* strain K1 (Obodozie et al., 2004). In another study **57** was isolated from a methanol stem bark extract of another plant from the same genus, *C. siamea*, and confirmed to have strong activity against multidrug-resistant *P. falciparum* K1 strain (IC_50_ of 5 μg/mL) (Ajaiyeoba et al., 2008). Such independent confirmation of the activity is obviously important but unfortunately rare, in part because such data are harder to publish.

The crude hexane and ethyl acetate extracts of the leaves of *Crateva adansonii* showed moderate anti-trypanosomal activity with a MIC of 12.5 μg/mL (Igoli et al., 2012). In a further study, purpurin-18 ethyl ester **(58)**, an anthraquinone derivative, was isolated from the extracts of the leaves of the related species *C. adansonii* and displayed a MIC of 6.25 μM against *T. brucei*. Molecular docking studies of **58** with some trypanosomal proteins revealed glutathione synthetase as its most likely target, followed by sterol-14α-demethylase and riboflavin kinase, with riboflavin kinase showing highest affinity (Igoli et al., 2014), although these models need experimental verification.

The methanol extract of the roots of *Abrus precatorius* was recently reported to possess antileishmanial activity with IC_50_ ± SD of 22.2 ± 0.54 μg/mL. The ethyl acetate fraction of the extract yielded two antileishmanial isoflavanquinones identified as abruquinone A **(59)** and abruquinone B **(60)**, which displayed identical activity against *L. major* (IC_50_ ± SD 6.35 ± 0.005 and 6.32 ± 0.008 μg/mL, respectively) and *L. tropica* (IC_50_ 6.29 ± 0.015 and 6.31 ± 0.005 μg/mL, respectively) (Okoro et al., 2020).

### Phenolics

Phenolics represent a diverse class of compounds whose structures contain at least one hydroxyl substituent on an aromatic ring. They include phenyl propanoids, benzoquinones, phenolic acids, acetophenones, phenylacetic acids, hydroxycinnamic acids, phenylpropenes, coumarins and isocoumarins, chromones, naphtoquinones, xanthones, stilbenes, anthraquinones, lignans, neolignans, lignins, and condensed tannins; and have been reported to have various medicinal properties including antioxidant, anticancer, anti-inflammatory, boosting immunity, etc. Phenolics are widely distributed in the plant kingdom.

**Figure 8:**
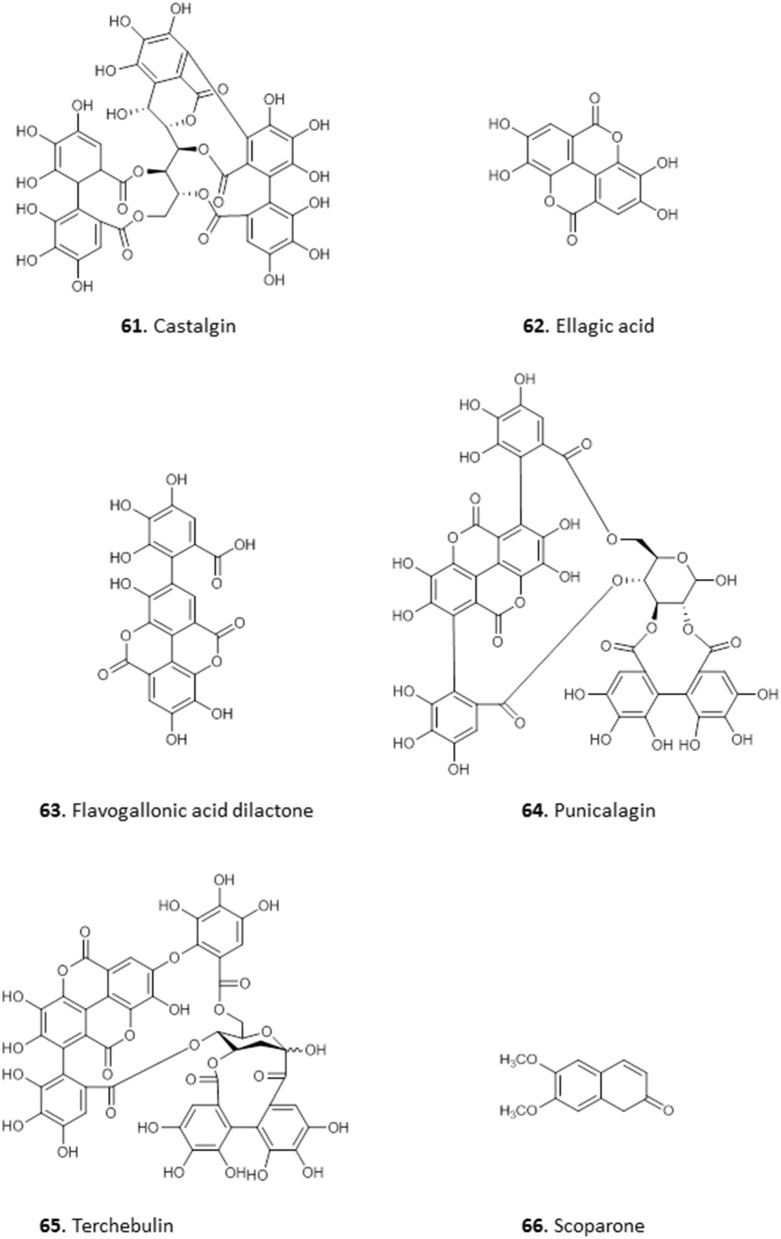
Structure of phenolic compounds isolated from Nigerian plants with selected antiprotozoal activity.

The antiprotozoal activity of the crude extract fractions and isolated tannins of *Terminalia avicennoides* and *Anogeissus leiocarpus* have been investigated along other Nigerian medicinal plants (Shuaibu et al., 2008a,b,c). Using PicoGreen as flourimetric monitor, the IC_50_ of the compounds against chloroquine-sensitive *P. falciparum* 3D7 was determined and all showed moderate activity: castalagin, **61** (10.57 μg/mL); ellagic acid, **62** (12.14 μg/mL); flavogallonic acid, **63** (8.89 μg/mL); punicalagin, **64** (9.42 μg/mL) and terchebulin, **65** (8.89 μg/mL). None of the compounds showed cross resistance to chloroquine (Shuaibu et al., 2008b). The compounds were also tested against trypanosome species *T. brucei brucei, T. b. ambience, T. b. rhodesiense*, and *T. evansi* and showed slightly higher activity against *T. b. brucei* with MICs of 22.5, 7.5, and 27.5 μg/mL for **61**, **63**, and **65**, respectively. However, the compounds displayed low antileishmanial activity with **61**, isolated from *Anogeissus leiocarpus*, being the most active with MIC of 55 μg/mL against *L. aethiopica*. Further morphological examination and electron microscopy revealed cell swelling and changes in the ultrastructure of organelles in *L. aethiopica* promastigotes exposed to **61** (Shuaibu et al., 2008a). All the tannins showed an MIC of ≥1,500 μg/mL against Newborn Mouse Heart Fibroblast cells in a cytotoxicity assay, suggesting good antiparasitic selectivity (Shuaibu et al., 2008a). The stem extract of *Euphorbia poisonii* yielded scoparone **66**, a coumarin with strong activity against *T. brucei* (MIC = 1.56 μg/mL) (Igoli et al., 2011) (for the structures of compounds **61**–**66**, see Figure 8).

### Glycosides

**Figure 9:**
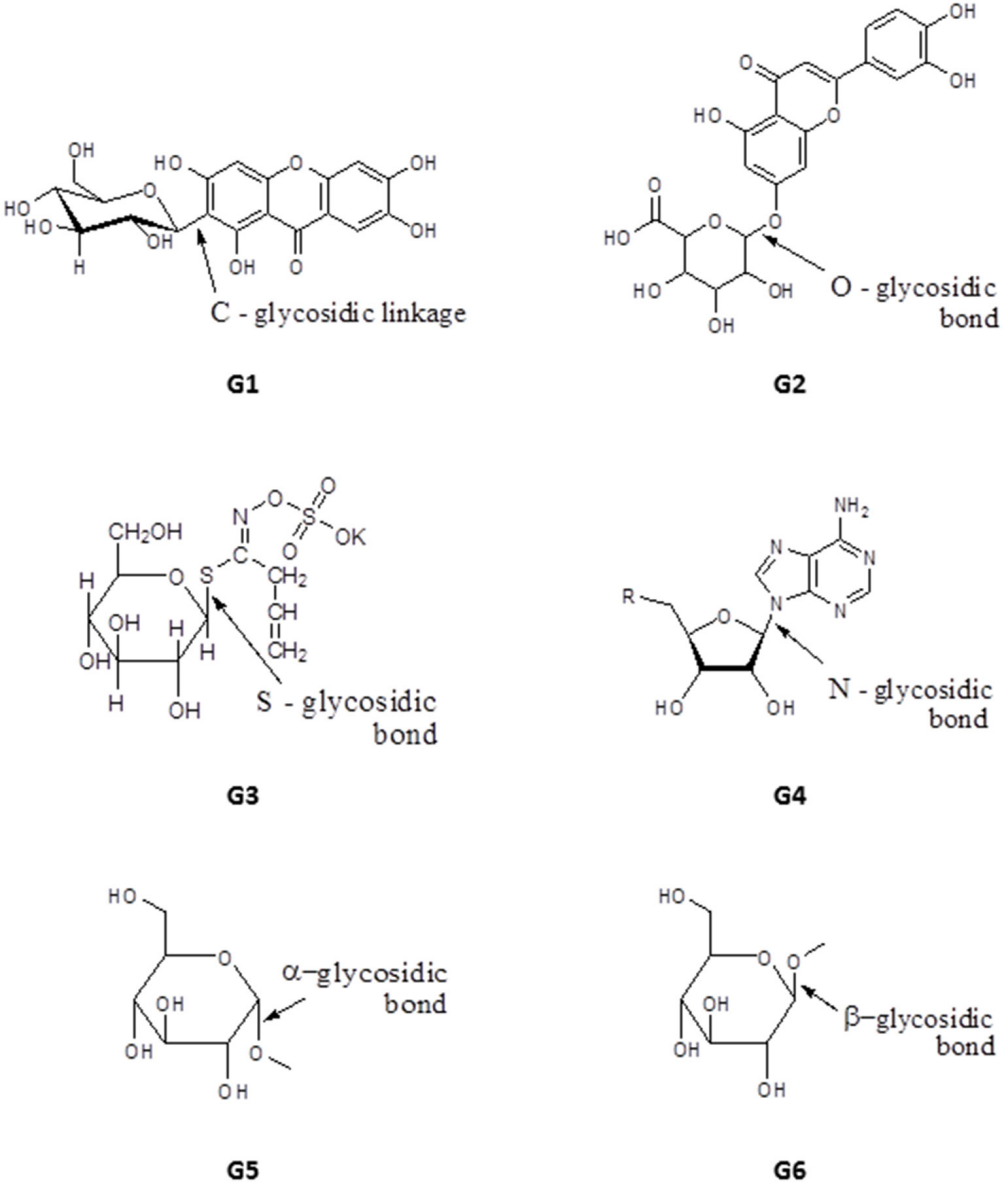
Structure of basic skeleton of glycoside sub-groups showing different glycosidic bonds.

Glycosides have one or more sugar moieties bonded through the anomeric carbon to a non-sugar group. The glycosidic bond can be carbon (C-glycosides, e.g., mangiferin, **G1**), oxygen (e.g., Luteolin-7-*O*-glucuronide, **G2**), sulfur (in thioglycosides e.g., Sinigrin, **G3**) or nitrogen (in glycosylamines e.g., adenosine, **G4**) (Figure 9). The sugar moiety is called the glycone and may contain one or more sugar groups, while the non-sugar group is the aglycone or genin part of the glycoside. According to their glycone moieties, glycosides are grouped into glucosides, fructosides, ribosides, glucuronides (glucuronnoside). Classification can also be based on the orientation of the glycosidic bond, α-glycosides have their glycosidic bond below the plane of the cyclic sugar moiety (glycone) e.g., methyl-α-Dglucopyranoside, **G5**, whereas when the glycosidic bond lies above the plane of the glycone a β-glycoside is formed e.g., methyl-β-D-glucopyranoside, **G6** (Figure 9). Classification of glycosides can also be based on their aglycone moieties e.g., steroid (cardiac) glycosides, coumarin glycosides, anthraquinone glycosides, saponins etc. The medicinal properties of glycosides have been exploited extensively for the treatment of heart diseases and as antibiotics, for example streptomycin, kanamycin and neomycin (Kren and Martinkova, 2001).

**Figure 10:**
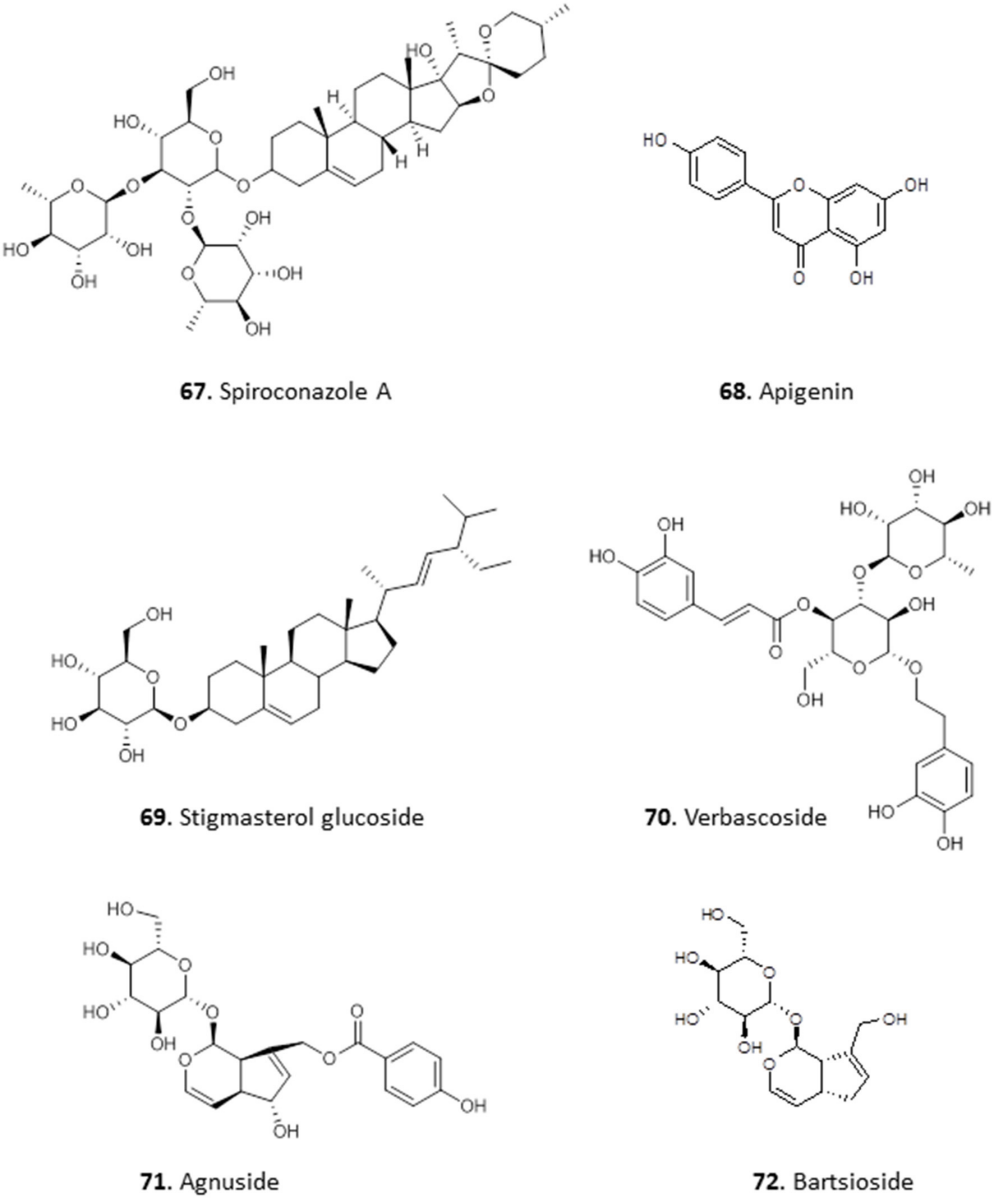
Structure of glycosides isolated from Nigerian plants with selected antiprotozoal activity.

Okunji et al. isolated a bioactive saponin, identified as Spiroconazole A (**67**), from the methanol extracts of the seed pulp of two “soap tree” species, *Dracaena mannii* and *D. arborea*. The compound showed significant antileishmanial and antimalarial activity (Okunji et al., 1996). Three glycosides were obtained from the methanol extract of the leaves of *Stachytarpheta cayennensis*, which is used in Central and West Africa to treat malaria. The compounds were identified as apigenin (**68**), stigmasterol glucoside (**69**) and verbascoside (**70**), and displayed significant (*P* < 0.05) antimalarial activity *in vitro* and *in vivo*, with a dose of 2.5 mg/Kg lowering *P. falciparum* parasitaemia in mice by up to 89% (Ifeoma Chinwude et al., 2016). Two antikinetoplastid irodid glucosides were isolated from the methanol leaf extracts of *Vitex grandifolia*. Agnuside (**71**) showed an IC_50_ of 5.38 and 13.7 μg/mL against *L. donovani* amastigotes in THP1 cells and against *T. brucei*, respectively. However, the other compound, bartsioside (**72**), was essentially inactive, with IC_50_ >25 μg/mL against both parasites, suggesting that the hydroxyl group on C-6 and/or the *p*-hydroxy benzoic acid moiety at C-8 are probably essential for activity (Bello et al., 2018) (for the structures of compounds **67**–**72**, see Figure 10).

### Peptides

Peptides are organic polymers made of amino acid monomers in which the alpha amino group (-NH) of one acid is linked to the alpha carboxylic acid group (-CO_2_H) of another through an amide bond. Peptides that contain more than 100 amino acid monomers are called proteins. Peptides have been isolated from roots, seeds, flowers, stems, and leaves of plants (Nawrot et al., 2014).

**Figure 11:**
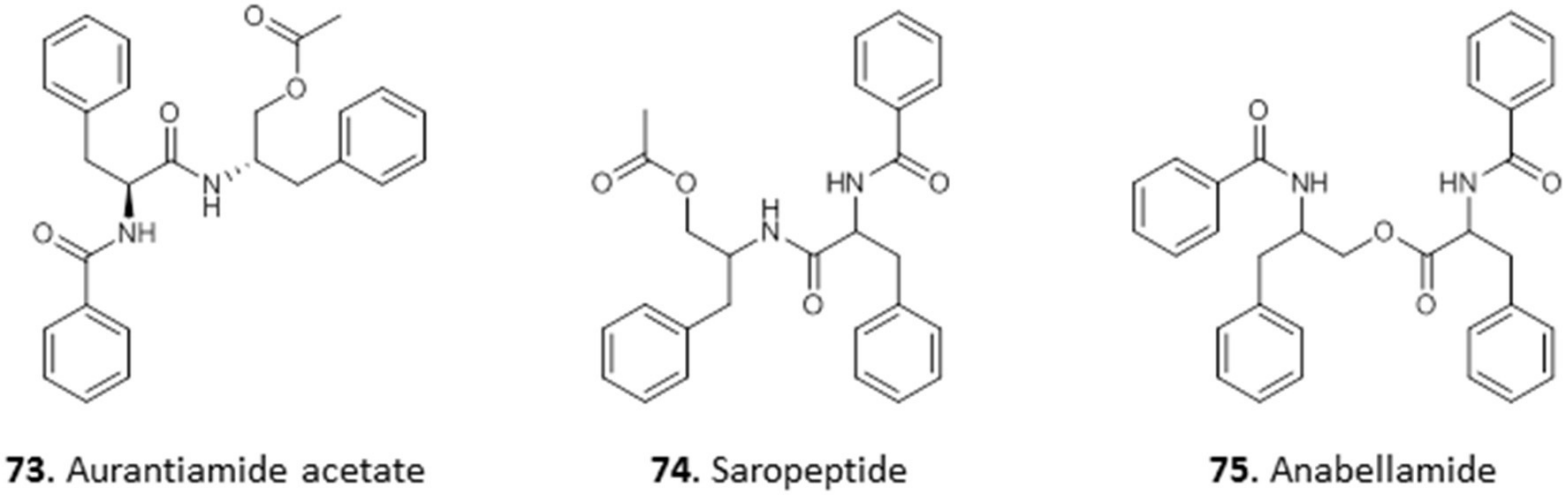
Structure of peptides isolated from Nigerian plants with selected antiprotozoal activity.

One antitrypanosomal peptide from Nigerian plants is aurantiamide acetate (**73**). It was isolated from the leaves of *Crateva adansonii*, and displayed an MIC of 25 μM against *T. brucei*. Further investigation into its possible mechanism of action through docking studies revealed strong binding interactions between **73** and trypanosomal enzymes sterol-14α-demethylase and trypanothione reductase (Igoli et al., 2014). Nwodo et al. (2015a) isolated two dipeptides from the methanol extract of the roots of *Zapoteca portoricensis* elucidated as saropeptide (**74**) and anabellamide (**75**). Compound **74** was selectively active against *T. brucei* with IC_50_ of 3.63 μM, but had only low activity against *Trypanosoma cruzi* (IC_50_ = 41.6 μM), which is the causative agent of Chagas disease. On the other hand, **75** displayed moderate activity against both species (*T. brucei* IC_50_ = 12.2 μM; *T. cruzi* IC_50_ = 16.1 μM). Although both compounds displayed relatively low toxicity against mammalian L6 cells with IC_50_ values of 92.1 μM and 71.2 μM for **74** and **75**, respectively, this does not seem to leave sufficient selectivity for further development (Nwodo et al., 2014) (for the structures of compounds **73**–**75**, see Figure 11).

### Fatty Acid and Steroids

**Figure 12:**
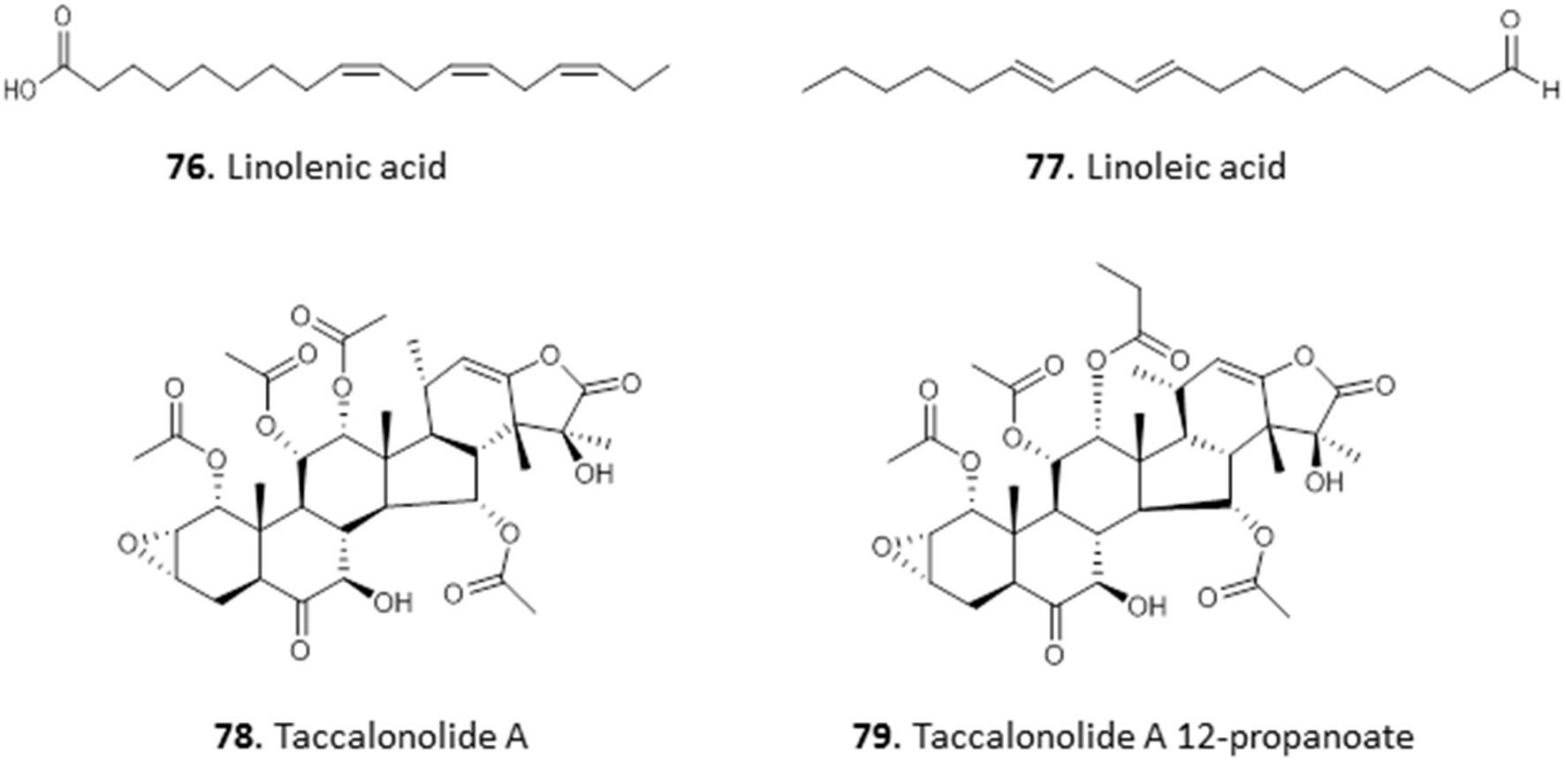
Structures of fatty acids and steroids isolated from Nigerian plants with selected antiprotozoal activity.

The ethyl acetate and methanol extracts of the leaves of *Carica papaya* exhibited promising antiplasmodial activity with IC_50_ values of 2.6 and 12.8 μg/mL in chloroquine-sensitive *P. falciparum* D10 strain, respectively. Bioassay-guided fractionation of the ethyl acetate extracts yielded two fatty acids, 9,12,15-octadecatrienoic acid (linolenic acid; **76**) and 9,12-octadecadienoic acid (linoleic acid; **77**) with IC_50_ values of 3.58 ± 0.22 and 6.88 ± 0.02 μg/mL in *P. falciparum*. The compounds were not cross-resistant to chloroquine but the level of selectivity for the parasite over Chinese hamster ovarian cells is quite low (SI = 7.43–15.27) and does not invite extensive *in vivo* follow-on (Melariri et al., 2011). In our own investigations of antiprotozoal natural products from Nigeria and beyond, we have reported the isolation of Taccalonolide A (**78**) and its novel derivative, Taccalonolide A 12-propanoate (**79**), from the tubers of *Tacca leontopetaloides*. Both compounds showed activity against *T. brucei*, with EC_50_ ± SEM of 11.4 ± 0.39 and 3.1 ± 0.09 μg/mL. However, we have observed remarkable antitrypanosomal activity in one of the impure fractions (EC_50_ = 0.76 ± 0.03 μg/mL), suggesting a need for further investigation of *T. leontopetaloides* as a source of more potent antiparasitic agents (Dike et al., 2016) (for the structures of compounds **76**–**79**, see Figure 12).

### Chlorophyll Metabolites

**Figure 13:**
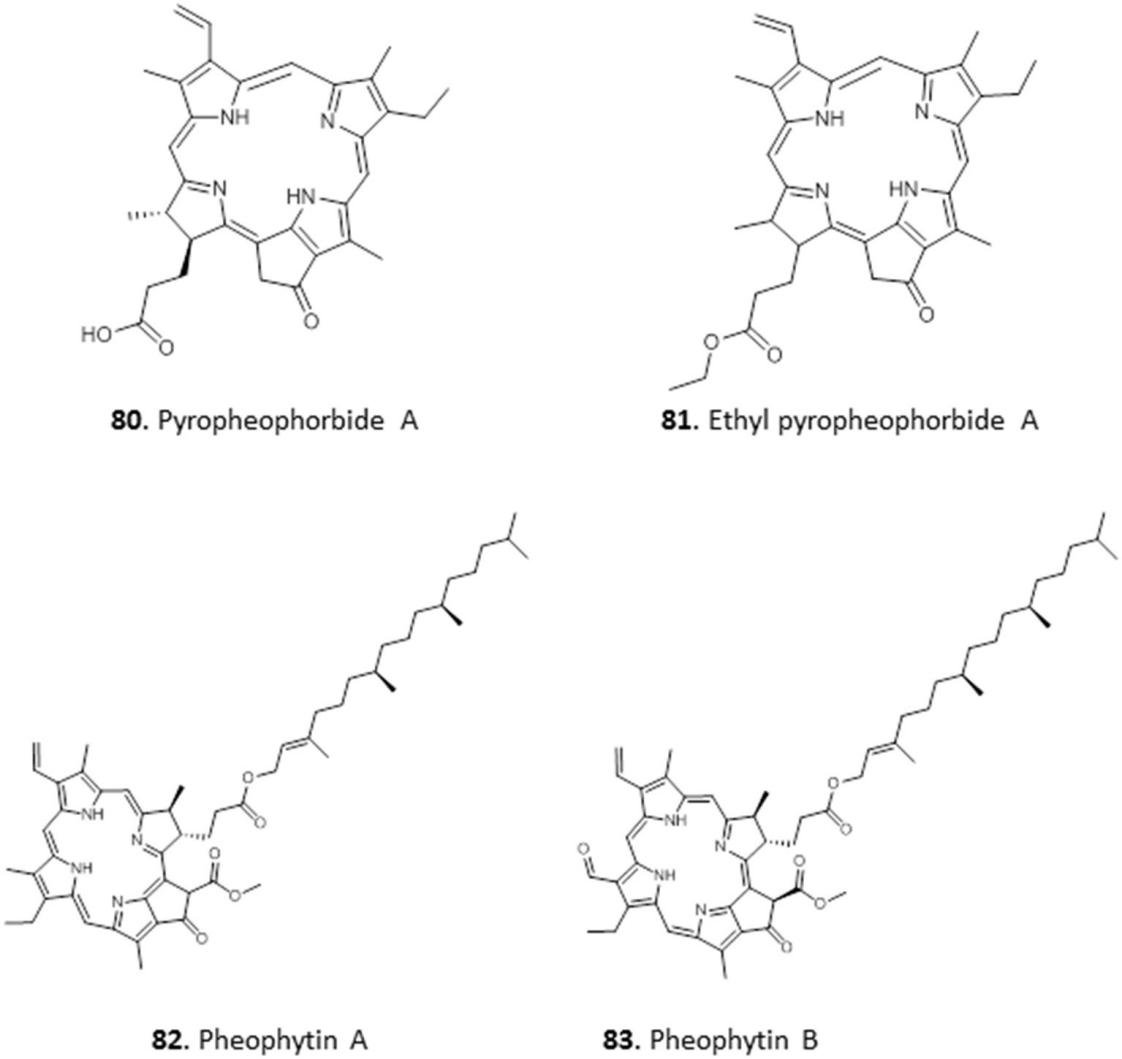
Structures of chlorophyll metabolites isolated from Nigerian plants with selected antiprotozoal activity.

Extracts of the leaves of *Crateva adansonii* has yielded two pheophorbides, Pyropheophorbide A **(80**) and Ethyl pyropheophorbide A **(81)**. Both **80** and **81** showed potent antitrypanosomal activity with an MIC of 6.5 μM. Both compounds showed strong binding interactions with trypanosomal riboflavin kinase and trypanothione reductase in molecular docking studies (Igoli et al., 2014). The ethyl acetate leaf extracts of *Newbouldia laevis* showed interesting activity against *T. b. brucei* (EC_50_ ± SEM = 4.2 ± 0.7 μg/mL). Two similar antitrypanosomal compounds, pheophytin A **(82)** and pheophytin B **(83)**, with EC_50_ ± SEM values of 25.0 ± 2.8 and 1.58 ± 0.09 μg/mL, respectively, were identified from the extract of *Newbouldia laevis* (Family Bignoniaceae); both compounds were non-toxic to HEK cells (Ebiloma et al., 2017) (for the structures of compounds **80**–**83**, see Figure 13).

## Compounds From Other Nigerian Natural Sources

### Lichen

A depside, 4-(1-hydroxylprop-1-en-1-yl) atranorin-1-carboxylic acid (**84**), was obtained from *Dirinaria picta*, a lichen epiphyte of *Elaeis guineensis* palm trees, collected in Port Harcourt, Rivers State. It showed some antiplasmodial activity with IC_50_ value of 37 μg/mL, and low toxicity to mammalian HeLa cells (IC_50_ = 100 μg/mL) (Afieroho et al., 2018).

### Nigerian Red Propolis

**Figure 14:**
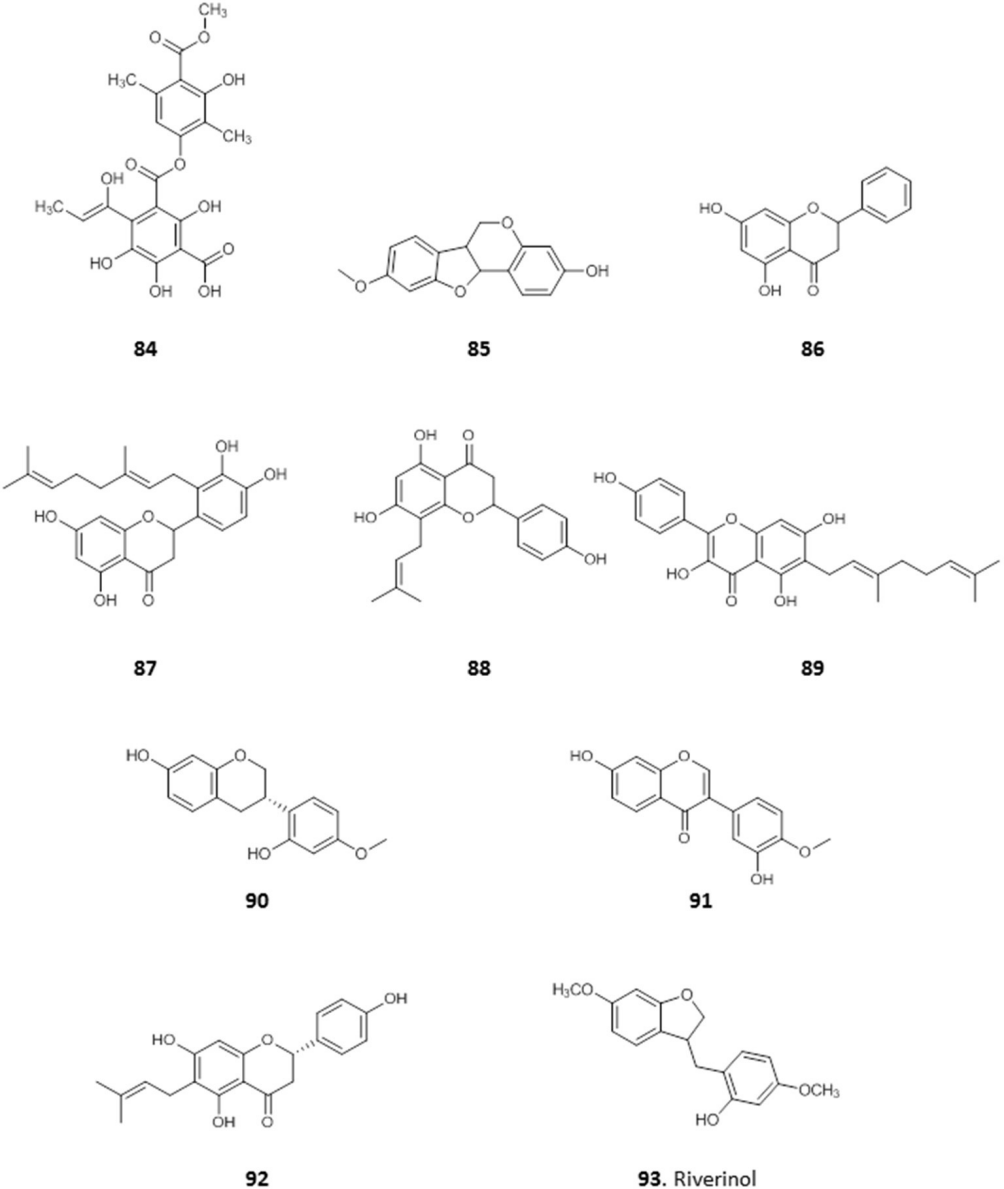
Structures of natural compounds isolated from Nigerian red propolis and lichen with selected antiprotozoal activity.

We have previously reported on the efficacy of Nigerian red propolis (NRP) in rats experimentally infected with *T. brucei*. Treatment with NRP at 400 and 600 mg/Kg/day resulted in significantly (*P* < 0.05) reduced parasitaemia and improved blood parameters (higher PCV and HBC) and weight gain compared to a DMSO treated control group (Nweze et al., 2017). This prompted further investigation into the active compounds in NRP. The ethanol extract of NRP yielded antitrypanosomal compounds, most of which were flavones and flavonoids (Omar et al., 2016). The MIC against *T. brucei* was determined for three of the compounds: medicarpin, **85** (3.12 μg/mL); pinocembrin, **86** (12.5 μg/mL), and propolin D, **87** (3.12 μg/mL) and EC_50_ values were determined for the remaining compounds. The most active of these were 8-prenylnarigenin (**88**), macarangin (**89**), and vestitol (**90**), with EC_50_ ± SEM of 6.1 ± 0.1, 7.8 ± 0.1, 8.3 ± 0.1 μg/mL, respectively. Other compounds with significant antitrypanosomal activity included calycosin (**91**; EC_50_ ± SEM = 10.0 ± 0.44 μg/mL), 6-prenylnaringenin (**92**; EC_50_ ± SEM = 11.4 ± 0.34 μg/mL), and riverinol (**93**; EC_50_ ± SEM = 16.24 ± 0.24 μg/mL). Interestingly, none of the compounds showed cross-resistance to pentamidine and melarsoprol but **93** was significantly more active against two multi-drug resistant strains (3.6 and 2.5-fold, respectively (*p* < 0.001) (Omar et al., 2016), from which either the TbAT1/P2 (Carter et al., 1999) or the HAPT1/AQP2 drug transporter (Alghamdi et al., 2020) had been deleted (for the structures of compounds **84**–**93**, see Figure 14).

## Barriers to Natural Product-Based Research and Drug Discovery

The challenges facing the development of new natural products-derived drugs discussed in this review are not peculiar to Nigeria alone but to many developing countries with richer natural resources than scientific infrastructure and funding. The identification of a pharmacologically active compound from a medicinal plant in a systematic and efficient manner is not an easy task. However, there is no gainsaying that specialized molecules of plant origin are the spine of our modern-day pharmacopeia, or that traditional herbal remedies continue to be successful in the treatment of many ailments. Notwithstanding this historic significance in drug discovery and development, there has been a steady drop in the number of new natural product-based drugs entering into clinical use in the past 30 years (Wright, 2019). The modern-day pharmaceutical industry has largely shifted from the traditional natural product-based drug discovery strategy to one that could easily be created with a synthetic approach such as combinatorial chemistry combined with high-throughput screening. Consequently, synthetic compounds are currently being favored by the global pharmaceutical industries over the natural product-based compounds as a more tractable alternative in their use to develop new drugs. This is a consequence of a combination of many factors.

### Complexity of Isolation Processes

Perhaps the most challenging impediments to natural product-based drug discovery over the past few decades are the resource intensiveness and the tedious purification protocols involved in the identification of bioactive molecules from the highly complex mixtures that are the initial extracts. When purified, the isolated bioactive compound is often obtained in tiny amounts (usually a few milligrams) that are unlikely to go the full length of the drug discovery process, especially in protocols involving the use of large cultures such as required for metabolomic studies and other assessments of drug action, or for *in vivo* studies. This is compounded by the fact that this tedious process frequently yields subversive compounds with multiple behavior in assays such as covalent bond formation, chelation, membrane perturbation, and redox activity, which are collectively known as Pan Assay Interference compoundS (PAINS) (Baell and Holloway, 2010; Baell, 2016). Prominent natural (plant) products that frequently contain PAINS include catechols, quinones, phenolic manic bases, and hydroxyphenylhydrazones (Baell, 2016). PAINS and PAINS-like, despite a possible nano- or micro-molar potency, lack a distinct biological mechanism, exhibit poor SAR or optimisability, and thus have very low prospects for clinical development (Baell, 2015). Thus, it is important to utilize assay techniques that will screen-out PAINS and to carry out structural studies of complexes of hits-target molecules and structure-activity optimization studies of the hits (Baell and Nissink, 2018; Balogun et al., 2019).

### Lack of Precision and Mechanism

In addition to the challenge of the tedious process flow from the screening of medicinal plants to the isolation of new bioactive principle(s) with low yield, the subsequent determination of the exact mechanism of action of the isolated compound requires expertise, experience, the appropriate research environment (infrastructure and equipment) and substantial amounts of funding. But due to research and resource limitations, finding a robust and viable lead candidate for the drug discovery pipeline has become a challenging scientific task. Hence, the majority of the research efforts aimed at identifying drug leads end up at the level of crude extract testing stage, which is not very informative and fails to give a usable measure of activity as the concentration of the active molecule(s) is unknown. Moreover, the amount of active compound in the extract is likely to be different between preparations.

In addition, the therapeutic effect of natural remedies may be through immuno-modulation rather than a direct antimicrobial activity. Assaying for antimicrobial properties, a compound with immunomodulatory properties will not show bioactivity *in vitro* during the traditional bioassay-guided screening of the complex extract mixture and will be ruled out. This is a serious impediment to determining the exact active principle in a medicinal plant or its extract unless the primary screen is based on an infectious animal model. Furthermore, some natural compounds may exhibit a combined or synergistic effect in their mode of action. Hence, such compounds, when isolated in their pure form, may not appear to have promising activity in the bioassay screening protocol (Druilhe et al., 1988; Williamson, 2001; Wagner, 2011). A prominent example is the case of the presently leading anti-malarial plant, *Artemisia annua*. Herbal teas prepared from the dried leaves of *A. annua* are found to be 6–18 folds more effective at killing the *Plasmodium* parasites than the isolated active anti-malarial agent, artemisinin (Wan et al., 1992; Wright et al., 2010). These possible scenarios can frustrate efforts toward identifying the bioactive compound(s) from some plants/extracts that show initial promise.

### Novelty of Isolated Natural Compounds

The majority of compounds isolated from plants will have been previously reported, and such compounds lacking novelty are unlikely to be advanced into drug development and less likely to lead to academic publications. In fact, over 200,000 isolated natural compounds have so far been reported in the scientific literature using the traditional natural product extraction and purification methods (Walsh and Yang, 2017). This means that there is a high probability of identifying known rather than novel molecules using the standard bioassays guided fractionation and identification of lead compounds, thus often giving a poor return on investment.

A range of processes known as “dereplication,” based on hyphenated techniques are standard procedures that are applied in plant metabolomics to avoid the re-isolation and re-characterization of known compounds by eliminating known entities (Carnevale Neto et al., 2016; Hubert et al., 2017; Kildgaard et al., 2017). However, to systematically extract vital pieces of information from acquired data, chemometric tools are needed for the dereplication process. This is because biological samples such as plant extracts are complex in nature, with a very large concentration range. This notwithstanding, the GC-MS-based technique for identifying non-targeted metabolites is one of the emerging techniques to improve the dereplication method (Carnevale Neto et al., 2016), but the procedures involved can be time-consuming and resource-intensive.

### Undesirable Physical Properties

Another fundamental challenge is that in the drug discovery setting, it is predicted that poor absorption becomes probable when there are more than 5 H-bond donors, 5 H-bond acceptors, the molecular weight of the compound is above 500, or the calculated partition coefficient (cLog P) is >5 (Lipinski et al., 1997). This is known as Lipinski's rule of five. Unfortunately, most natural compounds do not obey this rule; hence, in the eye of the pharmaceutical companies, many natural compounds do not have the required drug-like characteristics, based on their structure. This is in addition to the solubility issues associated with some natural compounds; many are not completely soluble in the assay's aqueous environment, which can interfere with the reproducibility of research results. Many of these issues require increased troubleshooting in standardized assays, leading to higher costs and a high rejection rate for otherwise active compounds.

Other undesirable physical properties of natural compounds like colouration make them more difficult to assay using contemporary drug discovery strategies such as the standardized high throughput screening of compound libraries, compared with synthetic compounds. For instance, deep colouration of some plant compounds may interfere with the assay methods involving color change in spectroscopic/colorimetric determinations.

Finally, it is imperative to highlight the huge structural complexities of many natural compounds, making synthesizing them and their derivatives very difficult (Harvey, 2000; Strohl, 2000; Li and Vederas, 2009), and the construction of a large series of related compounds for SAR or pharmacodynamic optimization almost impossible. The combination of these problems diminishes the prospects of using compounds of natural origins for drug discovery.

### Accessibility of Plant Materials and Poor Documentation of Herbal Remedies

Another significant challenge is the absence of specific government legislation and international agreements regulating the access to plant resources in the biodiversity-rich flora. Some of the indigenous plants used in folklore are rapidly disappearing due to indiscriminate use, deforestation, and deliberate bush-burning for farming activities and hunting. This makes subsequent access to these important medicinal plants for follow-up studies increasingly difficult, which in turn discourage further natural products research. This is a serious problem particularly in developing countries where the implementation of the Nagoya protocol is yet to be fully implemented. The Nagoya Protocol on Access and Benefit Sharing (ABS) is a build-up on the Convention on Biological Diversity (CBD), which is a key regulatory document for sustainable development aimed at developing national strategies for the conservation and sustainable use of biological diversity in all countries (Convention on Biological Diversity, 2010; Buck and Hamilton, 2011). Contrary to the opinions of critics of ABS who think it will create bottlenecks for scientific research and impedes new discoveries, the implementation of the Nagoya Protocol in Nigeria will prevent the extinction of plants from which useful medicinal products can be discovered. There can also be difficulty in physically gaining access to the natural habitats of these essential medicinal plants. This challenge is due to the absence of accessible roads to the often remote rural areas where the majority of these important medicinal plants are located.

Furthermore, traditional herbal remedies, which are justly regarded as the basis for modern medicine, are based on oral tradition (folklore) and much of that is usually shrouded in secrecy and a reluctance to divulge vital information regarding the preparation or use of the key medicinal plants. To keep the traditional healers in business, the pharmacologically active plants are often used in combination with other plants to prepare the herbal remedies, in order to obfuscate the true source of the medication. Unfortunately, but understandably, permission for the associated extracts/concoctions to be used for scientific research and innovation is frequently denied.

### Limited Research Funding

As explained above, the processes involved in the isolation, purification, and chemical characterization of the bioactive agents are expensive and time-consuming. Together with potential worries about continuous access to sufficient quantities of the natural compound, this makes the pharmaceutical industries less interested in investing in such ventures. This lack of investment is further compounded by the problem of reduced funding of academic research institutions, which hinders the provision of modern state-of-the-art facilities including such that enable long-term storage and (high-throughput) screening of natural products-based compound libraries. Scale-up of the production of natural compounds with complex (stereo)-chemistry that elude viable commercial synthesis can be another major hurdle, especially for compounds from relatively rare and/or slow-growing plants, or where very low or variable concentrations of active compounds are found in the primary plant source.

### Climate Change

Climate change has also already negatively impacted the natural product-based drug discovery effort. Environmental issues, like desert encroachment due to global warming, have led to the extinction of many valuable land species including key medicinal plants. In addition, there is the disappearance of marine organisms of high medicinal value due to water pollution and temperature increases.

These challenges can be overcome when there is a huge national, multidisciplinary approach to drug discovery efforts, and synergy between academia and pharmaceutical companies. This requires concerted efforts from the appropriate government ministries and parastatals, academia and research institutes, and the pharmaceutical companies. When the lack of resources is factored into this challenge, it is imperative to establish a close partnership between local research institutions and local or foreign pharmaceutical companies.

## Recommendations and Future Perspective

In this review we described and listed scores of phytochemicals isolated from Nigerian medicinal plants and, importantly, they have been reported to possess therapeutic capabilities against important Africa-endemic parasitic infectious diseases. The focus of this paper has been on the protozoan infections- malaria, African trypanosomiasis, and leishmaniasis. Apart from malaria, these diseases are popularly termed neglected tropical diseases (NTDs) because they disproportionately affect the economically bottom billion (world's poorest) people; consequently, drug discovery for such diseases is not of interest to the pharmaceutical industry due to projected lack of profitable return on the investments. Since the majority of the people affected by these diseases are Africans, it would be fulfilling, and indeed transformational, to see a few of the novel natural compounds that are highlighted in this review make their ways to the market as drugs of choice for malaria or some of the NTDs. It should be noted that, according to the World Health Organization, Nigeria is the country most afflicted by malaria, shouldering 25% of the global malaria burden (World Health Organization, 2019). In order words, the goal of this review is not only to provide a compendium of antiparasitic natural compounds from Nigerian bioresources, but also to stimulate and channel research focus toward further development of the compounds to marketable drugs. If successful, this will become a leading example of solutions to African problems from Africa, and by Africans. However, at this moment, every single compound listed here is still experimental and in the early stages of drug development. The subsequent paragraphs provide perspectives and steps to be taken toward genuine clinical development of African natural compounds for African diseases.

### Advancement of the Compounds Through the Drug Development Pipeline (DDP)

The process of drug development takes 7–15 years of hard work, huge funding, and is comprised of four stages each having multiple steps: (1) the discovery stage, involving successful demonstration of the compound as being potent against the target parasites; (2) the pre-clinical development stage where the compounds are tried in various laboratory animals to understand their safety, pharmacology, *in vivo* potency, and possible mechanism of action; (3) the clinical development stage, which is comprised of five phases of clinical trials; and (4) the approval stage where regulatory bodies evaluate the accumulated data of stages 1–3 and take a decision to approve or reject their use as medicines for specific applications. Certainly, some of the 93 compounds highlighted fulfill the *prima facie* criteria to move from stage 1 to stage 2, and considerations such as compound availability, ease of synthesis, selectivity, solubility, stability etc. would aid in prioritization. It is logical to explore a shortlist further and progress them through the DDP with the overall aim to get new approved medicine(s) for malaria and some NTDs, from the current collection of compounds, although further discovery efforts, coupled to innovative dereplication, remain important. To achieve this, and in order to enhance success within shorter time, it is recommendable that Nigerian scientists form interdisciplinary networks internally, with collaborators within as well as outside the continent, and with pharmaceutical companies and/or international non-profit organizations. One highly encouraging and very recent development is the partnership between the Drugs for Neglected Diseases initiative (DNDi), Institut Pasteur Korea (IPK) and Fundación MEDINA, funded by La Caixa Health Research [Drugs for Neglected Diseases initiative (DNDi), 2020]. This will allow the screening of the large natural compounds library of Fundación MEDINA against *Leishmania* and *T. cruzi* by IPK, using state-of-the art high-throughput imaging technologies. This development shows the added value of large compound libraries held and curated centrally, over the dispersed efforts of many small-scale efforts, when it comes to securing the needed funding and infrastructure. A national or, better, regional repository for medicinal plant species, extracts, and natural compounds should be established in Nigeria as a partner for international funders and consortia. While much excellent work has been done by the individual laboratories, results are not necessarily comparable between them, due to differences in test strains and procedures, among other factors. Nor are compounds produced this way, on a necessarily small scale and often sub-optimally stored, easily available for further experimentation. The proposed facility should be curated by a Society of Herbal Medicine, with State funding.

### Exploration of Microbes and Marine Organisms for More Novel Compounds

In addition to length of time needed, the attrition rate in DDP is very high. The number of starting compounds that enters the DDP drastically thins out after each step and stage- from available records only 2.5% of stage-1 compounds make it to stage 2, of which just 2% pass to stage 3, and 5% of the stage-3 candidates may eventually end up getting approved to be in the market (Kola and Landis, 2004; Tonkens, 2005). The implication is that only 1 out of 250 candidate compounds that enters stage 2 may become an approved medicine. With limited funding for even stage 2 studies, critical selection of what enters this stage is essential to decrease the attrition rate. Bearing in mind the aforementioned, it is advisable to continue natural compound discovery and screening against target pathogens, in a coordinated fashion, in order to expand this collection of compounds. Since most of the compounds at hand thus far are from plants, we are recommending that we expand our efforts to less-explored sources of medicinal natural products such as the microbial and marine organisms. Nigeria, and Africa at large, is blessed with diverse ecosystems that are habitats to myriad aquatic and non-aquatic macro- and micro-organisms. While the antibiotic penicillin is a leading example of drugs from microorganisms, the analgesic drug, Ziconotide was the first approved medicine from marine source. After these, numerous drugs from microbial and marine sources have been successfully brought to market (Molinski et al., 2009; Lobanovska and Pilla, 2017).

### Formulate a Virtual Library of the Compounds

The recent advances in the fields of medicinal chemistry, and computer and information technology have resulted in a synergy that birthed an emerging field known as computational medicinal chemistry, which basically entails *in silico* approaches for the curation, design, development, testing and synthesis of pharmacologically active compounds. *In silico* curation is simply the act of keeping a collection of information in computers and making it accessible to interested people remotely, this is simply a database or virtual library. This can be organized relatively easily and even after the establishment of a national repository as described above, would retain its relevance if actively curated and updated by the research community. Numerous relevant virtual libraries/databases are available containing information on parasites—e.g., for genomes PlasmoDB (https://plasmodb.org/plasmo/app), TriTrypDB (https://tritrypdb.org/tritrypdb/app); metabolism (http://vm-trypanocyc.toulouse.inra.fr/; Shameer et al., 2015) and small molecules, e.g., ChEMBL (https://www.ebi.ac.uk/chembl/), PubChem (https://pubchem.ncbi.nlm.nih.gov/), and the recently curated TrypInDB (http://trypindb.biomedinformri.com/; Vijayakumar et al., 2020). Likewise, we propose the formulation of a virtual library/database to contain the 93 highlighted antiparasitic natural products of Nigerian origin, as well as information on all tested but inactive natural compounds in order to minimize needless replication, and continue to expand the library as new pharmacologically active compounds are being discovered. This database should further be expanded in collaboration, first to include natural compounds reported from other African nations, and potentially from other continents. As the database will be freely accessible, it will promote collaborations with scientists within and outside the continent and accelerate the process of drug discovery. As it grows, it should be explored whether this database could be linked to other resources such as TrypInDB or CHEMBL in order to promote its visibility.

### Setting Up Synthesis Programs for the Various Compounds

Except for compounds **84**–**93**, which were isolated from Nigerian propolis, the remaining compounds (**1**–**83**) enumerated were isolated from plants (Table 1) and as expected, the final yields are very low. Environmental concerns and low yield are major hurdles for drug development efforts using natural products from plants because large amounts of compounds are usually required, particularly at stages 2 and 3. Moreover, even after approval of a final drug, it would be hard to envisage a good business model built on a product whose supply is reliant on its isolation from plants in very small quantities—especially since the cost per treatment for NTDs must necessarily be low. Thus, in many cases the crux of a profitable and sustainable drug development plan for plant products may be to be able to establish a process for its large-scale production independent of the native plant. Above, we have already highlighted the ease of synthesis as one of the shortlisting criteria for progressing natural compounds down the DDP, and efforts should be initiated to establish economically viable synthesis routes for the selected candidates. To the best of our knowledge, so far only compound **7** (fagaronine) has an established chemical synthesis procedure (Rivaud et al., 2012) and funding of natural compounds synthesis research should be part of the national scientific strategy of Nigeria.

Most of the natural compounds are structurally and stereochemically complex, making their chemical synthesis routes difficult, and often non-feasible in terms of cost/yield. For such compounds, the alternative to chemical synthesis will be synthetic biology. The use of the synthetic biology approach requires: (1) identification of the biosynthesis pathways and the genes encoding the enzymes for every step in the biosynthesis of the secondary metabolite (natural compound) in the native organism, (2) heterologous assemblage of the pathway in a suitable microorganism, which is achieved by cloning and transformation of all the genes into the surrogate microorganism, (3) establishment of a fermentation technology for the large-scale culture and production of the product, and (4) a standardized large-scale purification method for the produced secondary metabolite. Recently, the antimalarial terpenoid artemisinin, which is natively produced by the plant *Artemisia annua*, has been partially produced in yeast and *Escherichia coli* through synthetic biology, yielding quantities as high as 25 g/L of culture (Tsuruta et al., 2009; Paddon et al., 2013). Another interesting example of synthetic biology is the complete biosynthesis of noscapine in *Saccharomyces cerevisiae*. Noscapine is a potential anticancer drug that is natively derived from the opium poppy plant, *Papaver somniferum*. Engineering *S. cerevisiae* by introducing 10-gene clusters from opium poppy, which encodes 30 enzymes in the biosynthetic pathway of noscapine resulted in the heterologous production of the compound with a yield 2.2 mg/L (Li et al., 2018).

## Conclusion

Over the years, the bioactivity of extracts from Nigerian flora has been established, thus validating the widespread use of herbal medicine in that part of the world. Bioactivity-driven fractionation efforts have now identified candidate drug leads in some of these plants, including potential antiprotozoal agents. However, the number of pure compounds isolated and investigated is still low, despite the thousands of preliminary studies showing promising activity of plant extracts. Furthermore, the translation of *in vitro* anti-protozoal activity of the already identified phytochemicals into *in vivo* preclinical studies has also been slow. While the challenges affecting the development of these natural compounds are numerous and complex, they can be surmounted by concerted collaborative and multi-disciplinary efforts, especially when underpinned by a national strategy and the establishment of a natural compounds repository to enable it. This will facilitate competitive bids for international funding, enable synergy between academia and pharmaceutical companies and not-for-profit organizations, and would bridge many gaps in scientific infrastructure. Thus, while investment in research in developing countries like Nigeria is indispensable, coordination within the country is essential for the development of international collaborations. Nigeria has the raw materials and resources as well as the drive and the talent in their scientific community, now is the time to translate these into valuable pharmaceutics that benefit its population.

## Author Contributions

MU, JI, and GE conceptualized the project. MU, GE, HK, NI, and EB contributed to the development and writing of the manuscript. HK, JI, and EB contributed in validating, reviewing, and supervising the project. All authors contributed to the article and approved the submitted version.

## Conflict of Interest

The authors declare that the research was conducted in the absence of any commercial or financial relationships that could be construed as a potential conflict of interest.
